# Abrupt-mediated control of ninjurins regulates *Drosophila* sessile haemocyte compartments

**DOI:** 10.1242/dev.202977

**Published:** 2024-12-09

**Authors:** So Yeon Kwon, Kimberly Chan, Martin Stofanko, Ka Hei Chan, Paul Badenhorst

**Affiliations:** Institute of Cancer and Genomic Sciences, University of Birmingham, Edgbaston B15 2TT, UK

**Keywords:** *Drosophila*, Abrupt, Ecdysteroid, Ninjurins, Haemocyte adhesion

## Abstract

Macrophage-like cells called haemocytes are key effectors of *Drosophila* cellular innate immune function. Larval haemocytes exist either in circulation or localize to segmentally repeated sessile haemocyte compartments (SHCs). While numerous functions have been proposed for SHCs, the mechanisms directing haemocytes to them are unclear. Here, we have exploited the developmentally regulated dispersal of SHCs that occurs at pupariation to identify the Abrupt (Ab) transcription factor (TF) and ninjurin cell-adhesion molecules as regulators of haemocyte recruitment to SHCs. We show that larval haemocytes express ninjurins, which are required for targeting haemocytes to SHCs. However, at pupariation, ecdysteroid signalling stimulates Ab expression, which collaborates with TFs, including Blimp-1 and Hr3, to repress ninjurins and disperse haemocytes. We observe that experimental manipulations that antagonize ninjurin function in larval haemocytes cause premature SHC dispersal, while stabilization of ninjurins in haemocytes blocks developmentally regulated SHC remodelling and increases sensitivity to immune challenges. Cumulatively, our data indicate that control of ninjurin activity provides a common target through which diverse developmental, environmental and immune stimuli can be integrated to control haemocyte dispersal and immune function.

## Teaser

**Summary:** Control of ninjurin activity provides a common target through which diverse developmental, environmental and immune stimuli can be integrated to control haemocyte dispersal and immune function in *Drosophila*.

## INTRODUCTION

Macrophages are crucial mediators of the innate immune responses of both humans and invertebrates. In *Drosophila,* macrophage-like cells called haemocytes neutralize fungal and bacterial pathogens and parasites, participate in clearance of apoptotic cells during development, and are important mediators of wound healing and repair responses (reviewed by Mase et al., 2021; Wood and Martin, 2017). Haemocytes are generated during both embryonic and larval stages of development. Embryonic haemocytes derive from prohaemocyte precursors that originate from the procephalic or head mesoderm (Holz et al., 2003). These prohaemocytes differentiate into either ∼700 plasmatocytes (Tepass et al., 1994) or between 20 to 30 crystal cells (Lebestky et al., 2000). The crystal cells remain localized as bilateral clusters on either side of the embryo, while plasmatocytes disperse and follow a number of highly stereotyped migration pathways through the embryo (Bruckner et al., 2004; Cho et al., 2002; Tepass et al., 1994). Embryonic plasmatocytes persist into larval stages and increase by division, generating over 5000 plasmatocytes that constitute the bulk of haemocytes in circulation by the end of larval stages (Lanot et al., 2001).

In third instar larva, approximately two-thirds of haemocytes freely circulate in the haemolymph, while the remainder attach to the inner surface of the cuticle to form a number of segmentally repeated lateral and dorsal sessile accumulations that contain both plasmatocytes and crystal cells (Lanot et al., 2001; Makhijani et al., 2011; Stofanko et al., 2008). The function of these sessile haemocyte compartments (SHCs) is not entirely clear, although it has been proposed they act as defined haematopoietic compartments. Potential functions include providing a progenitor pool for lamellocytes and other cell types (Leitao and Sucena, 2015; Markus et al., 2009), acting as immune sentinels liberated upon infection (Stofanko et al., 2008) or, by analogy with mammalian tissue-resident macrophages, providing defined niches to drive functional specialization (Lavin et al., 2015). The lateral SHCs in particular are intimately associated with peripheral nervous system (PNS) neurons, and it is speculated that these provide cues that direct haemocyte localization or trophic support to maintain compartments (Makhijani et al., 2017, 2011). Similar sessile haematopoietic clusters have also been identified along the dorsal vessel of the adult (Ghosh et al., 2015; Sanchez Bosch et al., 2019). Understanding the mechanisms that control homing of haemocytes to SHCs and how targeting is regulated in response to developmental cues and immune stimuli are key to illuminating functions of these regions.

In previous work, we conducted a gain-of-function genetic screen to identify regulators of haemocyte development and homing (Stofanko et al., 2008). Candidates identified during this screen suggested that steroid hormone signalling acts to control haemocyte localization to SHCs. In *Drosophila*, ecdysteroids play a vital role in driving metamorphosis and development (reviewed by Thummel, 2001; Yamanaka et al., 2013). Pulses of ecdysteroids occur during embryonic and larval development, and orchestrate the major events of metamorphosis (Riddiford, 1993). A high-titre ecdysteroid pulse is observed at the end of the third-instar larval stage, which initiates the onset of pupariation (Lavrynenko et al., 2015). Changes in the adhesion of haemocytes to substrates have been observed before pupariation and after treatment with ecdysteroids both in *Drosophila* (Lanot et al., 2001) and Lepidoptera (Clark et al., 2005). Moreover, ecdysteroids have been shown to modulate haemocyte motility (Sampson et al., 2013), to influence haemocyte immune responses (Regan et al., 2013; Sorrentino et al., 2002) and to direct extensive remodelling of lymph glands at pupariation, allowing the liberation of lymph gland haemocytes into circulation (Grigorian et al., 2011).

In other developmental contexts the BTB (Broad-Complex, Tramtrack, Bric-à-Brac) and zinc-finger (ZF) transcription factor Abrupt (Ab) is a downstream mediator or modulator of steroid signalling. During ovarian border cell migration Ab integrates signals from the JAK/STAT and ecdysteroid signalling pathways, and is speculated to control ecdysteroid signalling via interactions with the ecdysteroid receptor co-activator Taiman (Tai) (Jang et al., 2009). Ab can also influence ecdysteroid signalling in the ovarian germline stem cell niche, where mutations in ecdysteroid pathway components and overexpression of Ab result in the formation of enlarged niches (Konig et al., 2011). Changes in Ab translation by the steroid-induced micro-RNA *let-7* has been shown to be required for the metamorphosis-induced switch of α/β neurons in the mushroom body of the *Drosophila* brain (Kucherenko et al., 2012).

Control of cell adhesion appears to be a common feature of Ab-regulated developmental processes. Ab regulates arborization of the dendritic arborization (da) neurons of embryos by controlling expression of the homophilic cell adhesion gene *Tenascin major* (*Ten-m*; Hattori et al., 2013). Similarly, in the mushroom body, Ab represses the cell adhesion molecule *Fasciclin 2* (*Fas2*) to provide a regulatory circuit through which steroid signalling orchestrates changes in cell adhesion by modulating Ab levels (Kucherenko et al., 2012). In embryos, Ab also regulates neuromuscular synapse formation, being expressed in muscles but required for motoneuron connectivity, suggesting it controls expression of neuronal targeting cues (Hu et al., 1995).

In this article, we show that dorsal SHCs undergo ecdysteroid-triggered remodelling with dispersal of haemocytes at pupariation. We exploit this developmentally regulated switch in haemocyte distribution to investigate Ab function in haemocyte targeting to and dispersal from SHCs. A key element of this work is the identification of the conserved homophilic cell-adhesion molecules ninjurins as transcriptional targets of Ab, and demonstration that ninjurins are previously unreported regulators of haemocyte adhesion, required normally for haemocyte recruitment to SHCs.

## RESULTS

### Larval dorsal sessile haemocyte compartments are remodelled at pupariation

Previous analyses have shown that the haemocytes progressively localize during larval development to segmentally repeated dorsal sessile haemocyte compartments (SHCs) (Stofanko et al., 2008; Zettervall et al., 2004). These are largely absent during the first larval instar but emerge during the 2nd larval instar, culminating in a stereotyped pattern of segmentally repeated dorsal SHCs in third larval instar stages (Stofanko et al., 2008; Stramer et al., 2005). Sessile haemocytes are lost at the end of the 3rd instar stage before pupariation (Lanot et al., 2001; Stofanko et al., 2008) in response to pulses of ecdysteroids that initiate metamorphosis and wholesale changes in body plan from larva to adult. We reasoned that such developmentally regulated remodelling of SHCs could provide a useful experimental system, both to identify factors controlling haemocyte dispersal from compartments, and to define and interrogate function of components that mediate homing to SHCs at earlier larval stages.

Before this model could be employed, however, we needed to be able to accurately stage 3rd instar larvae relative to pupariation, enabling the dynamics of SHCs to be mapped relative to the third larval instar ecdysteroid pulses. Moreover, detailed knowledge of the organization of the dorsal SHCs at larval stages, before dispersal, was also required to enable the identification of potential cues used to direct their formation. To stage larvae, we exploited changes in locomotor and feeding behaviour of 3rd larvae that occur in response to successive pulses in ecdysteroids (Riddiford, 1993). A small rise in 20-hydroxyecdysone (20E) occurs ∼30 h before pupariation (Lavrynenko et al., 2015) that initiates a switch in larval locomotor behaviour from foraging to wandering (Fig. S1A). This is followed by a larger surge in 20E that starts the transition to pupariation and that initiates the purging of the gut contents. By monitoring successive changes in locomotor behaviour from foraging to wandering, and by visualizing gut purging through adding dye into fly media, we could stage and segregate third instar larvae into three distinct age classes relative to pupariation. As shown in Fig. S1A-C, these are: (1) foraging larvae, which are 30 h or more away from pupariation with no ecdysteroid signalling; (2) wandering larvae, which are 12-24 h away from pupariation and experience the small rise in ecdysteroid levels; and (3) clear gut larvae, which are 4-6 h away from pupariation and experience the high titre ecdysteroid pulse that triggers the onset of pupariation.

**Fig. 1.**
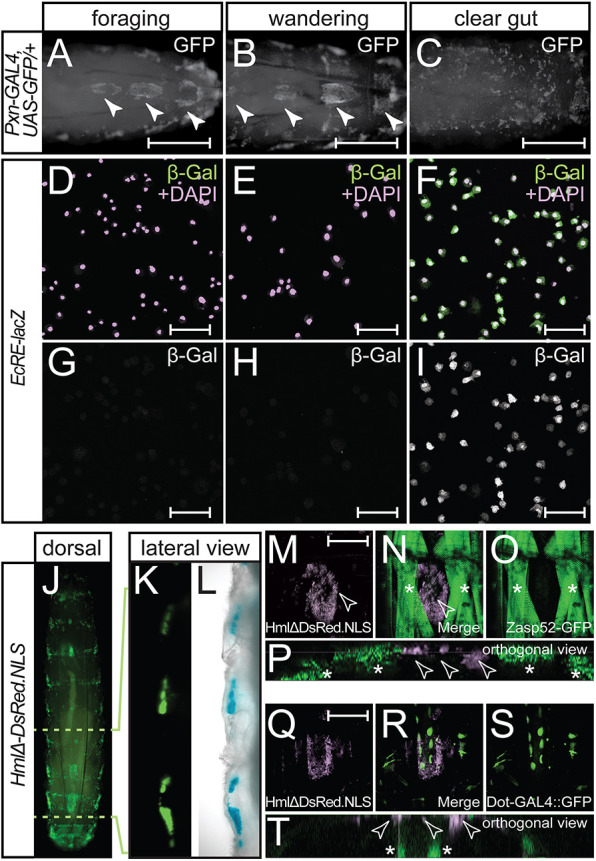
**Steroid regulation of dorsal sessile haemocyte compartments.** (A-C) Third instar larvae that express GFP under the control of the plasmatocyte- and crystal cell-expressed *Pxn-GAL4* driver were staged relative to the late larval ecdysteroid pulse and dorsal SHCs (closed arrowheads) visualized in (A) foraging stage, (B) wandering stage and (C) clear gut stage third instar larvae. (D-I) Anti-β-galactosidase staining of haemocytes isolated from (D,G) foraging stage, (E,H) wandering stage and (F,I) clear gut stage *EcRE-lacZ* third instar larvae. In merge images (D-F), anti-β-galactosidase staining is shown in green and DAPI-stained nuclei are in purple. (J-L) Live imaging of *HmlΔ-DsRed.NLS* 3rd instar larvae reveals that sessile haemocytes occupy compartments located immediately under the dorsal epidermis. (J) Dorsal view of entire *HmlΔ-DsRed.NLS* larva showing segmentally repeated SHCs. (K,L) Magnified lateral view showing (K) dsRed alone or (L) merge with differential interference contrast view. (M-P) Imaging of larvae containing both *HmlΔ-DsRed.NLS* reporter and GFP protein trap in the muscle Z band gene *Zasp52* (*Zasp52-GFP*) shows that sessile haemocytes (open arrowheads) are flanked by DO1 and DA1 muscles (asterisks). (Q-T) Imaging of larvae containing both *HmlΔ-DsRed.NLS* reporter and pericardially expressed GFP (*Dot-GAL4, UAS-mCD8-GFP*) reveals that sessile haemocytes (open arrowheads) are flanked ventrally by the dorsal vessel and pericardial cells (asterisks). Scale bars: 500 µm in A-C; 50 µm in D-I; 200 µm in M-T.

Deploying these staging methods with larvae that express GFP under the control of the haemocyte-expressed *Peroxidasin-GAL4* (*Pxn-GAL4*) or *Hemolectin-GAL4* (*Hml-GAL4*) drivers, we were able to visualize dorsal SHCs in live third instar larvae at different timepoints across the third instar ecdysteroid pulses. We observed that haemocytes accumulated in prominent dorsal SHCs in both foraging or wandering stage third instar larvae (arrowheads in Fig. 1A,B). However, these distinct dorsal SHCs were lost at the clear gut stage third instar larvae when haemocytes disperse (Fig. 1C).

By isolating haemocytes from larvae that contain an ecdysteroid-responsive reporter (*EcRE-lacZ*) (Koelle et al., 1991) we could monitor ecdysteroid signalling in haemocytes at these three larval stages and correlate it with SHC disruption. Immunostaining using antibodies against β-galactosidase (β-Gal) showed no expression of the *EcRE-lacZ* reporter in foraging stage haemocytes (Fig. 1D,G). A slight, but statistically significant, increase in anti-β-Gal staining occurred in wandering stage haemocytes (Fig. 1E,H, Fig. S2A), consistent with the small rise in ecdysteroids that initiates wandering behaviour. However, this was dwarfed by robust signal in clear gut stage haemocytes (Fig. 1F,I, Fig. S2B), indicating that loss of dorsal SHCs in clear gut larvae was accompanied by significant activation of ecdysteroid signalling in haemocytes.

We next used live imaging of larvae that express nuclear-localized DsRed in haemocytes (Makhijani et al., 2011), either alone or in combination with other fluorescent markers to characterize organization of the dorsal SHCs before dispersal. By tracking haemocyte nuclei in SHCs at the wandering stage, we were able to quantify compartment haemocyte number, determining that the largest posterior SHC contains up to 1500 haemocytes in wandering stage larvae (Fig. 1J-L, Fig. S3). Moreover, imaging of *HmlΔ-DsRed.NLS* larvae that also express either a muscle marker (*Zasp52-GFP*) (Fig. 1M-P) or a pericardial cell marker (*Dot-GAL4::UAS-mCD8-GFP*) (Fig. 1Q-T) demonstrated that dorsal SHCs occupy defined niches that are flanked laterally by dorsal acute and oblique muscles (DO1 and DA1; Balakrishnan et al., 2021) from each larval hemi-segment (Fig. 1M-P), and ventrally by the dorsal vessel (Fig. 1Q-T). Unlike, lateral SHCs (Makhijani et al., 2017, 2011), dorsal SHCs do not appear to be associated with PNS neurons (data not shown).

### Abrupt regulates SHC dispersal at pupariation

**Fig. 2.**
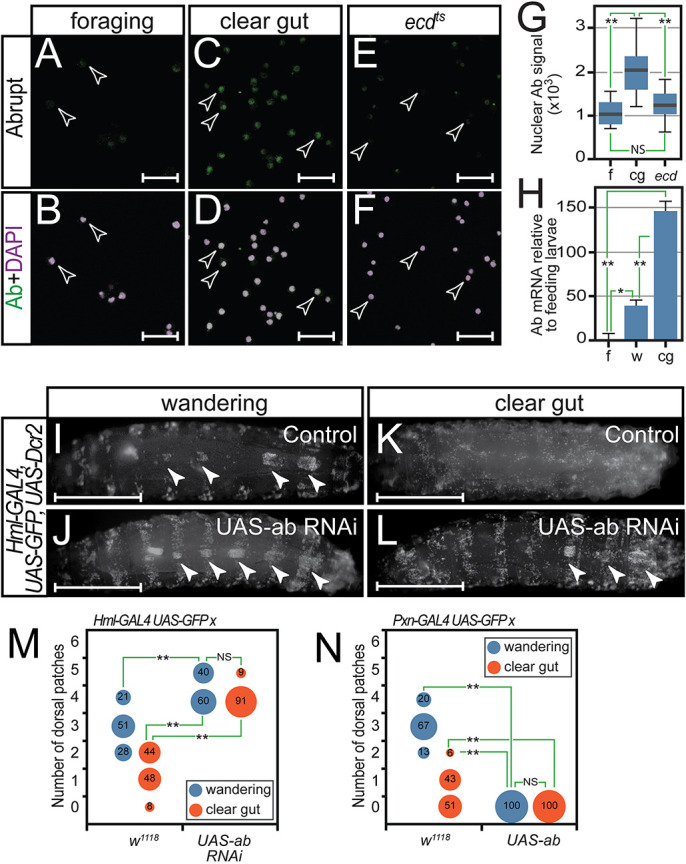
**Abrupt haemocyte expression increases are coincident with disruption of sessile compartments.** (A-F) Anti-Ab antibody staining of haemocytes from (A,B) foraging stage *w^1118^* third instar larvae, (C,D) clear gut stage *w^1118^* third instar larvae and (E,F) clear gut equivalent *ecd^1^* larvae raised at the restrictive temperature. Ab protein is shown in green in merge. Scale bars: 50 µm. (G) Quantitation of nuclear Ab signal intensities confirms significantly elevated Ab in clear gut (cg) relative to foraging (f) stage haemocytes, and a statistically significant reduction in Ab levels in clear gut stage *ecd^1^* haemocytes (ecd). (H) Real-time PCR analysis of *ab* transcripts levels in foraging stage (f), wandering stage (w) and clear gut stage (cg) larval haemocytes. *RpL32* was used as a normalization control. (G,H) For boxplots, the horizontal line represents the median, box limits represent the Interquartile Range (IQR) between first and third quartiles, and whiskers extend to the most extreme data points within 1.5× the IQR from the first and third quartiles. A one-way ANOVA test revealed a significant difference between the means for G (*F*=77.804, p<0.001) and H (*F*=151.041, *P*<0.001). Dunn's post-hoc tests were conducted to test pairwise comparisons: **P<0.001; *P<0.01; NS, not significantly different (*P*=0.261). (I-L) Comparison of SHCs in (I,K) *Hml-GAL4, UAS-GFP, UAS-Dcr2* driver larvae and (J,L) *UAS-ab* RNAi-expressing larvae shows that Ab knockdown stabilizes dorsal SHCs (closed arrowheads). Scale bars: 1 mm. (M,N) The number of dorsal SHCs was quantitated in at least 50 larvae of control driver and respective (M) *UAS-ab RNAi* knockdown and (N) *UAS-ab* overexpressing larvae at both wandering and clear gut stages. Distribution of SHC numbers is plotted as a bubble graph. A Kruskal–Wallis test revealed a significant difference in the means for knockdown (H=326.494, *P*<0.001) and overexpression (H=331.21, *P*<0.001). Dunn's post-hoc tests of pairwise comparisons: **P<0.001; NS, not significantly different in M (*P*=0.28) and N (*P*=1.0). Data in I-N were generated contemporaneously with data in Fig. 4A-H using a shared control group for each driver.

We previously isolated the transcription factor Ab in a gain-of-function genetic screen to identify regulators of third instar haemocyte patterning (Stofanko et al., 2008). These Ab overexpression phenotypes, together with other reports showing that ecdysteroid signalling can regulate Ab expression and control expression of cell adhesion molecules (Kucherenko et al., 2012), prompted us to investigate whether Ab similarly controls haemocyte dispersal from dorsal SHCs at pupariation. Consistent with Ab regulating haemocyte dispersal, anti-Ab staining showed that nuclear Ab was absent in foraging stage haemocytes (Fig. 2A,B) but detected in clear gut stage haemocytes when they disperse (Fig. 2C,D). Differences in Ab expression were confirmed by quantitation of nuclear Ab signal, with clear gut stage haemocytes exhibiting statistically significantly increased Ab signal (Fig. 2G).

Increased Abrupt protein levels were due to increased *ab* transcription, as real-time PCR analysis of transcript levels (Fig. 2H) indicated 140-fold higher *ab* mRNA expression in clear gut larval haemocytes relative to foraging stage haemocytes. In addition, we confirmed that ecdysteroid signalling was required for increased Ab expression by performing anti-Ab staining on haemocytes isolated from *ecdysoneless* (*ecd^1^*) mutant larvae in which ecdysteroid signalling is abrogated. Although wild-type clear gut stage haemocytes exhibit nuclear Ab, this was absent from *ecd^1^* mutant haemocytes of the equivalent stage (Fig. 2E-G).

We further demonstrated, using inducible RNA interference (RNAi), that Ab was required in haemocytes for SHC remodelling at clear gut stages. *ab* knockdown in haemocytes resulted in expansion of dorsal SHCs in wandering stage larvae (compare control and knockdown in Fig. 2I and J) and retention in clear gut larvae when they are normally disrupted (compare control and knockdown in Fig. 2K and L); this was confirmed by statistical analysis of dorsal SHC numbers across multiple larvae of each stage and genotype (Fig. 2M). In contrast, Ab overexpression in larval haemocytes before the normal onset of Ab expression resulted in the complete loss of SHCs at all stages examined (Fig. 2N), demonstrating that Ab was both necessary and sufficient to initiate SHC dispersal.

### Abrupt regulates expression of ninjurin cell-adhesion molecules

**Fig. 3.**
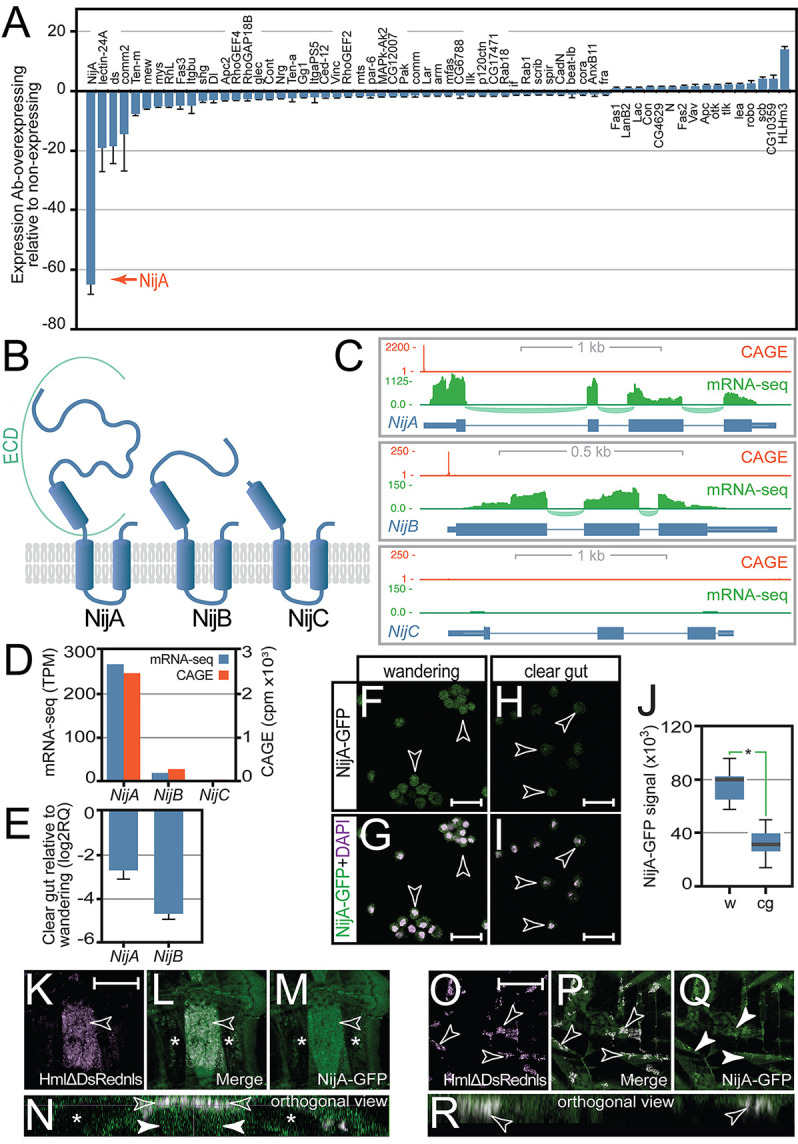
**Abrupt regulates expression of the homophilic cell-adhesion molecules NijA and NijB.** (A) Real-time RT-PCR analysis of a panel of 63 cell-adhesion molecules in haemocytes after Ab overexpression identifies NijA as an Ab gene target. (B) Membrane topology prediction indicates that NijA and its paralogues NijB and NijC adopt a shared conformation with a variable N-terminal extracellular domain (ECD), a core transmembrane domain of two membrane-spanning alpha helices, with a short, C-terminal extracellular domain. (C,D) Analysis of third larval instar CAGE-seq and mRNA-seq datasets using (C) a genome browser view and (D) a plot of normalized tag counts demonstrates robust expression of *NijA*, weak expression of *NijB* and absence of *NijC* in larval haemocytes. (E) Real-time RT-PCR comparison of wandering and clear gut stage larval haemocytes shows both *NijA* and *NijB* are downregulated at pupariation. (F-I) Anti-GFP antibody staining of (F,G) wandering stage and (H,I) clear gut stage *NijA-GFP* gene trap third instar larvae revealed that NijA protein levels (green in merge) decrease coincident with increased ecdysteroid signalling and loss of SHCs at pupariation. Scale bars: 25 µm. (J) Quantitation of NijA-GFP signal intensities confirms significantly reduced NijA-GFP in clear gut larvae. For boxplots, the horizontal line represents the median, box limits represent the Interquartile Range (IQR) between first and third quartiles, and whiskers extend to the most extreme data points within 1.5× the IQR from the first and third quartiles. *P=7.46891E-17 (unpaired *t*-test). (K-N) Live imaging of larvae containing both the *HmlΔ-DsRed.NLS* reporter and *NijA-GFP* reveals that dorsal sessile haemocytes (open arrowheads) express NijA and are associated with non-haemolytic tissue that expresses NijA (closed arrowheads). Asterisks indicate flanking dorsal muscles. Scale bar: 200 µm. (O-R) Lateral sessile haemocytes (open arrowheads) similarly express NijA and are located on top of tissue that expresses NijA (closed arrowheads). Scale bar: 200 µm.

In other developmental contexts, Ab regulates expression of cell-adhesion molecules that include Fas2 (Kucherenko et al., 2012) and Ten-m (Hattori et al., 2013). The remodelling of SHCs we observed at pupariation, or after ectopic Ab expression in larvae, was consistent with altered haemocyte adhesion. To discriminate targets of Ab in haemocytes, we determined the effects of Ab overexpression in haemocytes on expression of a panel of previously identified Ab targets, along with additional genes associated with cell adhesion, as defined by their Gene Ontology (GO) biological process and molecular function classifications. As shown in Fig. 3A, the most significant effect observed after Ab overexpression in haemocytes was a 98% reduction in expression of the homotypic cell-adhesion molecule *Ninjurin A* (*NijA*). Although Ab overexpression repressed *Ten-m*, no effect was observed on *Fas2*.

NijA is part of a conserved family of homophilic cell-adhesion molecules, of which the first identified member, Nerve injury-induced protein 1 (Ninjurin 1, Ninj1) was detected as a factor upregulated after nerve injury in rodents (Araki and Milbrandt, 1996). *Drosophila* possess three ninjurin orthologues: *NijA*, *Ninjurin B* (*NijB*) and *Ninjurin C* (*NijC*). All share homology to mammalian ninjurins within a central hydrophobic, membrane-spanning ninjurin domain (Fig. S4A, Pfam PF04923) flanked by variable N-terminal and C-terminal extracellular domains (Fig. 3B). *Drosophila* ninjurins display significant diversity in the extent of the N-terminal extracellular domain, with NijA displaying an extracellular domain that constitutes half of the protein, while NijC lacks extensive extracellular domains and NijB is intermediate between these extremes (Fig. 3B). Both mammalian Ninj1 and fly NijA have been confirmed to act as homophilic cell-adhesion molecules (Araki and Milbrandt, 1996; Zhang et al., 2006) that can be regulated by matrix metalloprotease cleavage of their ectodomains (Jeon et al., 2020; Zhang et al., 2006).

### Haemocyte expression of ninjurins

To discriminate which of the *Drosophila* ninjurin isoforms, or combination of isoforms, play a role in haemocyte targeting, we first determined expression levels of *NijA, NijB* and *NijC* in wandering stage larval haemocytes. Expression was monitored both by CAGE-seq (Cap analysis of gene expression sequencing) to discriminate and monitor the activity of transcription start sites (TSSs), as well as by mRNA-seq to determine splice variants. This analysis indicated robust expression of *NijA* in haemocytes, 90% lower expression of *NijB* and no expression of *NijC* (Fig. 3C,D). As only *NijA* and *NijB* were expressed in haemocytes, we focussed further study on these*,* examining first whether their expression was downregulated in haemocytes when SHCs are disrupted. Real-time RT-PCR comparison of *NijA* and *NijB* transcript levels between wandering and clear gut stage haemocytes showed that both were downregulated in clear gut haemocytes, at the point when sessile patches are dispersed (Fig. 3E). Similarly, anti-GFP antibody staining of haemocytes from *NijA-GFP* third instar larvae showed that while NijA-GFP protein could be detected in wandering stage haemocytes (Fig. 3F,G), signal was absent from clear gut haemocytes (Fig. 3H,I), confirmed by quantitation of NijA-GFP signal (Fig. 3J).

Ninjurins have been shown to function as homophilic cell-adhesion molecules and to cause cells to aggregate (Araki and Milbrandt, 1996; Zhang et al., 2006). We speculated that this activity is harnessed by haemocytes to trigger both the initiation and maintenance of SHCs. Initially, localized expression of ninjurins in non-haemocyte tissues that underlie future dorsal SHCs could be responsible for the specific targeting of ninjurin-expressing haemocytes to these regions. Once recruited, continued haemocyte expression of ninjurins could enable clustering of haemocytes within dorsal SHCs to maintain these structures. To provide evidence for this, we performed live imaging of *HmlΔ-DsRed.NLS* larvae that also contain the *NijA-GFP* gene-trap. This revealed that NijA expression could be detected in haemocytes that comprise the dorsal sessile compartment (Fig. 3K-N, open arrowheads) but was also observed in tissue that underlies these haemocytes (Fig. 3N, closed arrowheads). NijA expression was not detected in the flanking DO1 and DA1 muscles. Similarly, lateral SHCs were also observed both to express NijA and be associated with NijA-expressing non-haemocyte tissue (Fig. 3O-R). Taken together, these results suggest that localized ninjurin expression in non-haemocyte tissue may facilitate haemocyte targeting to establish SHCs.

### Ninjurins control sessile compartment formation

**Fig. 4.**
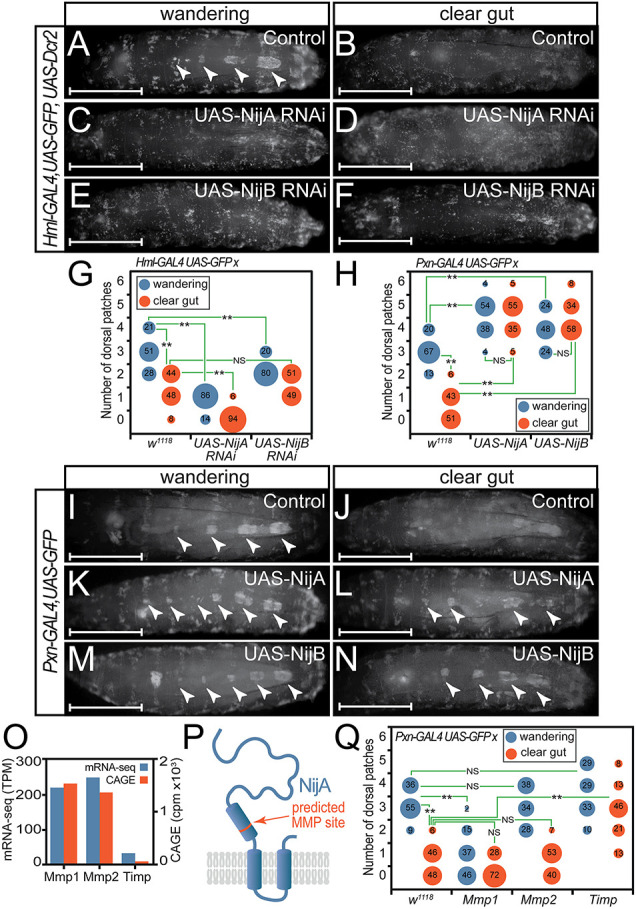
**NijA and NijB are required for dorsal sessile haemocyte compartment formation.** (A-F) SHCs in (A,B) *Hml-GAL4, UAS-GFP; UAS-Dcr2* driver alone control, (C,D) *NijA-* and (E,F) *NijB*-knockdown 3rd instar larvae. (G) Numbers of SHCs were quantitated after *NijA* and *NijB* knockdown in at least 50 larvae of each genotype at wandering and clear gut stages. A Kruskal–Wallis test indicated a significant difference between means (H=467.217, p<0.001). Dunn's post-hoc tests were conducted to test pairwise comparisons: **P<0.001; NS, not significantly different (*P*=0.255). (H) SHC numbers were quantitated in larvae after haemocyte-specific overexpression of NijA or NijB relative to *Pxn-GAL4, UAS-GFP* driver alone crossed with *w^1118^*. A Kruskal–Wallis test indicated a significant difference between means (H=409.076, *P*<0.001). Pairwise comparisons using Dunn's post-hoc tests identified significant differences: **P<0.001; NS, not significantly different [*P*=0.896 (UAS-NijA) or *P*=0.22 (UAS-NijB)]. (I-N) SHCs in (I,J) *Pxn-GAL4, UAS-GFP* driver alone larvae and in larvae with haemocyte overexpression of (K,L) NijA or (M,N) NijB. Arrowheads indicate SHCs. (O) Normalized CAGE-seq and mRNA-seq read counts reveals robust expression of Mmp1 and Mmp2 but low expression of Timp in third larval instar haemocytes. (P) Schematic of NijA, demonstrating location of a predicted MMP cleavage site between extracellular domain and transmembrane domain. (Q) Haemocyte-specific overexpression of Mmp1 disrupts SHCs while Timp overexpression increases SHC number. Mmp2 overexpression has little effect. *Pxn-GAL4, UAS-GFP* driver alone crossed with *w^1118^* provides the control. A Kruskal–Wallis test showed a significant difference between means (H=328.574, *P*<0.001). Dunn's post-hoc tests were conducted to test pairwise comparisons: **P<0.001; NS, not significantly different [*P*=0.126 (clear gut UAS-Mmp1 versus control); *P*=0.979 (clear gut UAS-Mmp2 versus control); *P*=0.640 (wandering UAS-Mmp2 versus control); *P*=0.158 (wandering UAS-Timp versus control)]. Data in A-H were generated contemporaneously with data in Fig. 2I-N using a shared control group for each driver. Data in Q were generated contemporaneously with data in Fig. 6E using a shared control group. Scale bars: 1 mm in A-F,I-N.

To confirm that ninjurins are required for the establishment and maintenance of dorsal SHCs, we experimentally altered ninjurin levels in haemocytes by knockdown or overexpression, and determined consequences on dorsal SHCs at both wandering and clear gut stages. As shown in Fig. 4A-F, knockdown of *NijA* and *NijB* resulted in the early loss of dorsal patches in wandering stage larvae, replicating phenotypes observed after Ab overexpression and confirming that ninjurins are required to maintain dorsal SHCs. Comparison of the distribution of dorsal compartment numbers across multiple larvae of each stage and genotype demonstrated that disruption was greater after *NijA* than *NijB* knockdown (Fig. 4G), indicating that NijA plays a dominant role in regulating larval haemocyte adhesion, consistent with the greater abundance of *NijA* relative to *NijB* transcripts in haemocytes. In contrast, the GAL4-mediated overexpression of NijA or NijB that counteracts their normal repression at pupariation by Ab led to the expansion of dorsal SHCs in wandering stage larvae (compare Fig. 4I with Fig. 4K and Fig. 4M), as well as their retention in clear gut larvae when they are normally disrupted (compare Fig. 4J with Fig. 4L and Fig. 4N), confirmed by comparison of the distribution of dorsal compartment numbers across multiple larvae of each stage and genotype (Fig. 4H).

Requirements for ninjurins were further confirmed by exploiting the ability of matrix metalloproteinases (MMPs) to cleave and therefore perturb ninjurin function (Jeon et al., 2020; Zhang et al., 2006). *Drosophila* contains two MMPs (Mmp1 and Mmp2), of which only Mmp1 interacts with ninjurins (Zhang et al., 2006), cleaving NijA between the extracellular and transmembrane domains (Fig. 4P), and disrupting NijA-mediated haemocyte adhesion. Consistent with this, *Pxn-GAL4-*mediated overexpression of Mmp1, but not Mmp2, induced the loss of dorsal SHCs at wandering stages (Fig. 4Q), replicating NijA loss-of-function phenotypes. In contrast, overexpression of the sole *Drosophila* tissue inhibitor of metalloproteinases (Timp; Wei et al., 2003), which should prevent NijA cleavage and stabilize NijA-mediated haemocyte adhesion, increased SHCs at wandering stages and stabilized SHCs at clear gut stages (Fig. 4Q), mimicking NijA-overexpression phenotypes.

### Chromatin profiling shows direct regulation of NijA by Ab

**Fig. 5.**
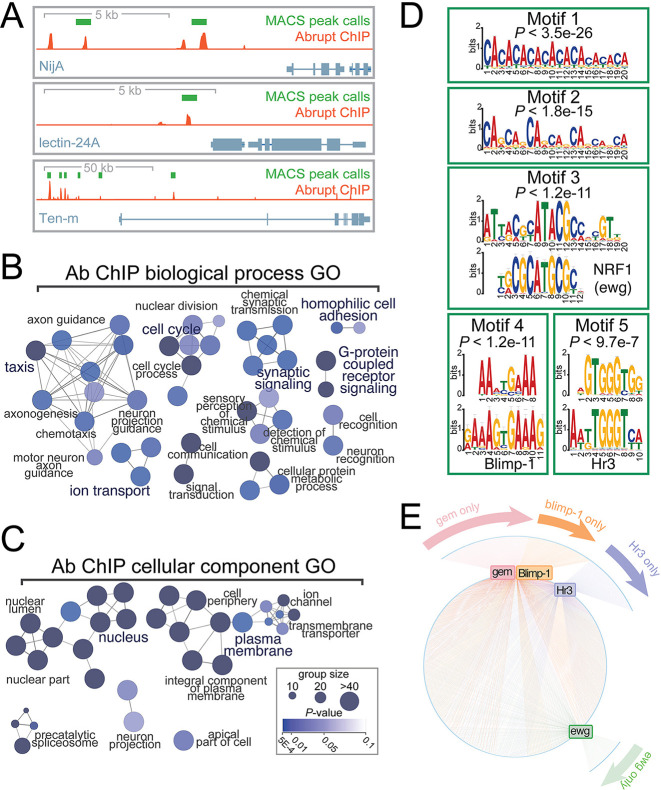
**Whole-genome mapping of Ab targets in late larval haemocytes.** (A) Distribution of Ab ChIP-seq signals on transcriptional targets (orange trace). Genes are oriented in a 5′→3′ direction with high-confidence MACS2-called peaks indicated in green. (B) Gene ontology (GO) network analysis of Ab ChIP targets according to biological process. (C) GO network analysis of Ab ChIP targets according to cellular component. Key indicates colouring according to statistical-significance of GO term enrichment and size indicates the number of genes in each node for B and C. (D) 250 bp DNA sequences flanking high-confidence Ab ChIP-peak summits were used for motif discovery and enrichment using Xstreme (MEME) to detect putative Ab DNA-binding consensi. Significantly enriched motifs together with matches (identified by TOMTOM) with known TFs are displayed. (E) iRegulon analysis of the 5 kb upstream cis-regulatory regions of Ab ChIP target genes identified statistically significant enrichment of known DNA-binding sites for the fly TFs Gem, Ewg, Hr3 and Blimp-1 in the regulatory regions of Ab target genes. For each identified TF, network analysis was performed to show the extent of overlap (and co-regulation) in Ab targets between each TF. Individual TFs are displayed as rectangular nodes coloured according to TF identity (Gem, pink; Ewg, green; Hr3, purple; Blimp-1, orange). Individual Ab target genes are displayed as blue ovals around the perimeter of the circle and regulatory connections (scored by enrichment of binding sites) between TFs and targets are displayed as edges (lines) coloured according to the TF. Targets that are regulated by more than one TF lie on the circle; those that are regulated by a single TF lie outside the circle and are labelled according to the single TF that binds the regulatory region.

Collectively, our results demonstrate that ninjurins are both necessary and sufficient for the establishment and maintenance of SHCs in larvae, but that Ab expression before pupariation represses NijA to cause SHC dispersal. To demonstrate that Ab repression of NijA is direct, and to identify other Ab targets in haemocytes, we performed chromatin immunoprecipitation sequencing (ChIP-Seq) on clear gut larval haemocytes using anti-Ab antibodies (Hu et al., 1995). As shown in Fig. 5A, this analysis detected robust Ab peaks on upstream regulatory regions of *NijA,* confirming direct regulation of *NijA* by Ab. Additionally, this analysis confirmed direct regulation of Ten-m and lectin-24A, genes we have previously demonstrated to be repressed by Ab overexpression in haemocytes By determining the genes located nearest to high confidence Ab ChIP peaks, we were able to identify an expanded set of Ab target genes in haemocytes. Consistent with Ab controlling haemocyte adhesion, these exhibit significant enrichment in GO biological process (Fig. 5B) and in cellular localization terms (Fig. 5C) for adhesion, taxis, signalling and membrane localization.

**Fig. 6.**
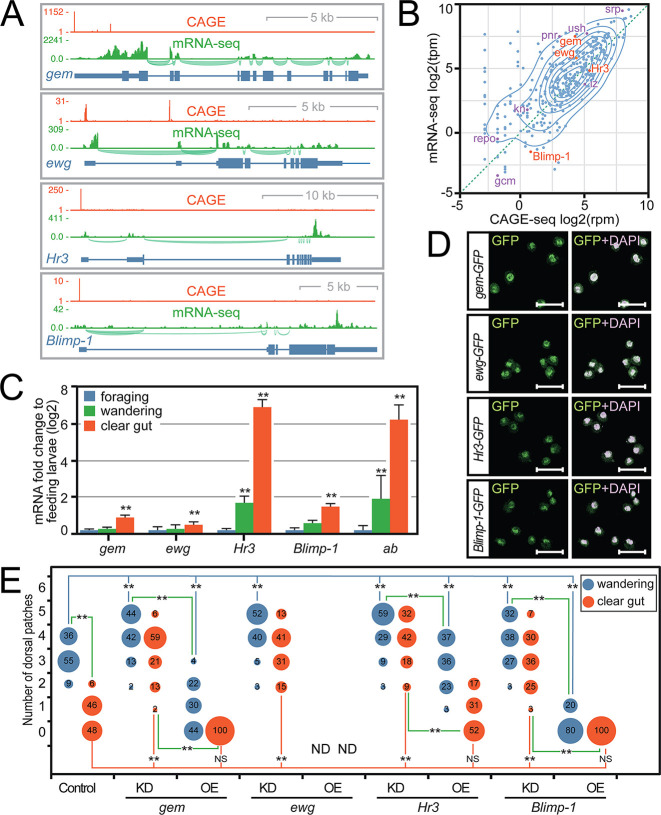
**Ab co-factors identified by ChIP also regulate haemocyte SHC remodelling.** (A) CAGE-seq and mRNA-seq analysis of TF co-regulators confirms expression in larval haemocytes. (B) Plot of normalized third larval instar haemocyte mRNA-seq versus CAGE-seq read counts for all known *Drosophila* TFs. TFs identified in this study (*gem*, *ewg*, *Hr3* and *Blimp-1*) are highlighted in orange with previously-identified haemocyte TF regulators (*srp*, *pnr*, *ush*, *lz*, *gcm* and *repo*) shown in purple. (C) Real-time PCR analysis of TF transcripts levels in foraging, wandering and clear gut stage larval haemocytes reveal increased expression of *gem*, *ewg*, *Hr3* and *Blimp-1* in haemocytes before pupariation. *RpL32* was used as a normalization control. **P<0.05 indicates values statistically significantly different from foraging stage (Student's unpaired *t*-test). Data are mean±s.d. (D) Anti-GFP staining of clear gut haemocytes isolated from C-terminal GFP-tagged *gem-GFP*, *ewg-GFP*, *Hr3-GFP* and *Blimp-1-GFP* strains. In merge panels, anti-GFP staining is shown in green and DAPI-stained nuclei in purple. Scale bars: 25 µm. (E) SHC numbers were analysed in larvae after either haemocyte-specific inducible dsRNA-mediated knockdown (KD) or overexpression (OE) of *Blimp-1*, *ewg*, *gem* and *Hr3* relative to control *Pxn-GAL4, UAS-GFP* driver alone. ND for *ewg* overexpression indicates that expression results in loss of GFP expression in haemocytes, both in SHCs and in the circulation. A Kruskal–Wallis test showed significant difference between the means (H=693.211, *P*<0.001). Dunn's post-hoc tests were conducted to test pairwise comparisons: **P<0.001; NS indicates not significantly different [*P*=0.450 (clear gut UAS-gem versus control); *P*=0.915 (clear gut UAS-Hr3 versus control); *P*=0.382 (clear gut UAS-Blimp-1 versus control)]. Data in E were generated contemporaneously with data in Fig. 4Q using a shared control group.

By collating the DNA sequences underlying Ab ChIP peaks and performing *de novo* motif discovery, we confirmed that the most highly enriched motifs recovered in our ChIP peaks (Fig. 5D, motif 1 and 2) matched previously published Ab-binding consensi (Turkel et al., 2013), confirming that ChIP peaks detected reflect genuine Ab-binding events. However, motif analysis of Ab ChIP peaks, as well a complimentary motif search of the 5 kb upstream cis-regulatory regions of putative Ab ChIP target genes, also identified additional motifs for TFs that bind alongside Ab and could be part of the regulatory network that controls haemocyte adhesion. These included the fly TFs Erect wing (Ewg) (Fig. 5D, motif 3), Blimp-1 (Fig. 5D, motif 4), Hr3 (Fig. 5D, motif 5) and Gemini (Gem) (Fig. 5E).

### Validation of a TF network that collaborates with Ab to control dorsal SHCs

To determine whether Gem, Ewg, Hr3 and Blimp-1 function alongside Ab to control SHC remodelling at pupariation, we characterized their expression and localization in haemocytes relative to dorsal SHC remodelling. CAGE-seq and mRNA-seq analysis of expression alongside that of known haemocyte TF regulators, such as Srp, Ush and Lz, demonstrated that all were expressed in late larval haemocytes (Fig. 6A) and at equivalent levels to TFs known to function in haemocytes (Fig. 6B). Using transgenic strains that express Gem, Ewg, Hr3 and Blimp-1 as C-terminally-tagged GFP fusion proteins, we demonstrated that they are both translated and localized to the nucleus in clear gut haemocytes (Fig. 6D), confirming function. Moreover, by analysing expression in haemocytes across third instar larval stages, from foraging to clear gut stages, we were able to show that expression of *gem, ewg, Hr3* and *Blimp-1* mirrors that of *ab*, with significant upregulation in clear gut stages at the point when SHCs are remodelled (Fig. 6C).

Most importantly, by using haemocyte-specific RNAi, we demonstrated that all were necessary for dorsal SHC remodelling. Knockdown of *gem, ewg, Hr3* or *Blimp-1* stabilized dorsal SHCs at the clear gut stage (Fig. 6E, compare Cont with KD panels), recapitulating the phenotypes observed after *ab* knockdown or NijA overexpression. Reciprocally, overexpression of these TFs (except for *ewg*, which could not be assayed) disrupted dorsal SHCs, with the strongest effects observed with Blimp-1 (Fig. 6E, compare Cont with OE panels). Significantly though, overexpression phenotypes were milder than Ab overexpression phenotypes, suggesting that Ab plays the dominant role in triggering haemocyte dispersal.

### Deregulation of dorsal SHC dispersal impairs responses to immune challenge

In the final part of the work, we investigated whether regulation of the release of haemocytes from SHCs is required for larval immune responses. It has previously been speculated that dorsal SHCs provide a reservoir where haemocytes are held latent in the absence of immune challenge but from which they can be deployed into circulation to deal with challenge (Stofanko et al., 2008; Zettervall et al., 2004). We therefore examined whether interfering with the release of haemocytes into circulation from SHCs, by overexpressing NijA in larval haemocytes, would affect survival in response to immune challenge. Three immune stimuli were assayed: the response to sterile wounding, the response to septic wounding with Gram-negative bacteria (*P. entomophila*) and the response to septic wounding with Gram-positive bacteria (*E. faecalis*).

**Fig. 7.**
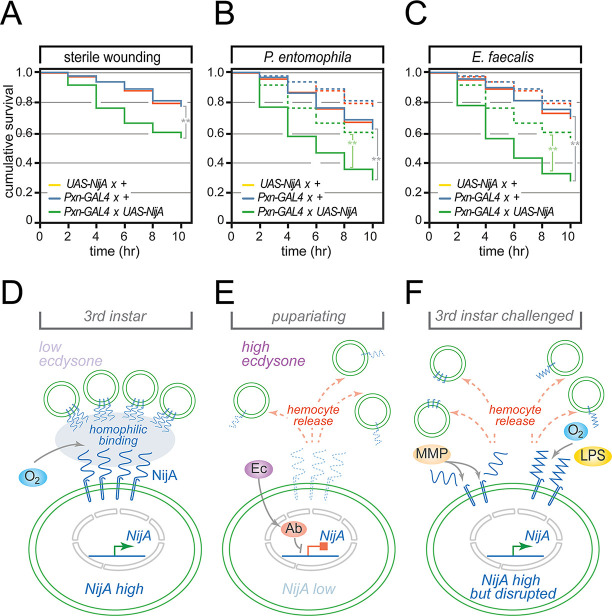
**Stabilization of SHCs by NijA overexpression decreases inflammatory responses.** (A-C) Haemocyte-specific overexpression of NijA decreases 3rd larval survival relative to *Pxn-GAL4, UAS-GFP* driver or *UAS-NijA* responder alone in response to (A) sterile injury and acute bacterial infection with (B) *P. entomophila* (Gram-negative bacteria) and (C) *E. faecalis* (Gram-positive bacteria). For A-C, mean survival was calculated from five cohorts of 30-40 larvae per genotype or condition assayed every 2 h. Genotypes used were: *Pxn-GAL4, UAS-GFP* driver alone (blue), *UAS-NijA* responder alone (orange) and *Pxn-GAL4, UAS-GFP×UAS-NijA* (green). Dashed lines in B and C indicate survival levels of sterile wounded larvae of each genotype for comparison. Results of pairwise comparisons (Log Rank (Mantel-Cox) are indicated: **P<0.001. Grey bars indicate comparisons between NijA over-expressors and control lines for each wounding condition; green bars indicate comparisons between sterile-wounded and septic-wounded NijA over-expressing larvae. (D-F) Models for the function of ninjurins in controlling haemocyte targeting to SHCs. (D) In the absence of immune challenge and before pupariation, when ecdysteroid (Ec) levels are low, NijA expression is high and facilitates haemocyte clustering and targeting to non-haemocytic tissue that express NijA. (E) During late larval stages, ecdysteroid (Ec) levels rise, increasing Ab to repress *NijA*, leading to haemocyte dispersal. (F) Alternatively, at stages where Ec levels are low and *NijA* is still high, immune challenge and/or inflammation can lead to NijA ectodomain cleavage by matrix metalloproteases (MMPs) or disruption (e.g. by oxygen tension or LPS, as observed with human Ninj1) that disperses haemocytes.

We observed statistically significant reductions in survival of larvae overexpressing NijA in haemocytes in response to all these challenges. These ranged from a small, but significant, reduction in survival in response to sterile injury in NijA-overexpressing larvae (Fig. 7A), to much greater reductions in survival after septic injury with either *P. entomophila* (Fig. 7B) or *E. faecalis* (Fig. 7C). Statistically significant reductions in survival were observed after septic injury for NijA-overexpressing larvae relative to driver/responder alone controls or relative to NijA-overexpressing sterile wounded larvae (Fig. 7B,C). Cumulatively these results suggest that preventing the release of sessile haemocytes impairs larval responses to immune challenge, implying that control of SHC dispersal is required for a full immune response.

## DISCUSSION

The wholesale reorganization of the *Drosophila* body plan that occurs at pupariation is accompanied both by differentiation of new tissues as well as extensive remodelling of larval structures that are repurposed and persist into pupal and adult stages. The paradigm for such re-orchestration has been the ecdysteroid triggered developmental pruning of neurons during pupariation (Truman, 1990). However, larval haemocyte populations undergo similar developmental remodelling, with larval haemocytes contributing vital functions to immunity and tissue remodelling during pupal stages (Lanot et al., 2001; Stephenson et al., 2022). Here, we show that larval dorsal SHCs undergo ecdysteroid-triggered dispersal before pupariation. This transition is orchestrated by rising levels of the Ab TF that represses ninjurin cell-adhesion molecules that are required for haemocyte adhesion. We demonstrate that overexpression of Ab leads to loss of dorsal SHCsn while *ab* RNAi leads to apparent expansion of these sessile haemocyte domains. In contrast, overexpression of NijA stabilizes these compartments, while knockdown leads to loss of sessile patches. Cumulatively, this defines a trajectory by which ecdysteroids control Ab expression that, in turn, represses ninjurins to disperse SHCs.

Developmental control of haemocyte adhesion by Ab is consistent with data observed in other tissues that indicate that Ab has a widespread role in regulating tissue-specific cell adhesion molecules (Diaz-Benjumea and García-Bellido, 1990; Hu et al., 1995; Kucherenko et al., 2012; Li et al., 2004). The developmental remodelling of SHCs we observe is one example that reflects a larger programme of tissue re-organization that occurs throughout the larval body during the transition to pupariation. We have shown that dorsal SHC remodelling requires Ab, acting in concert with additional TFs, including Gem, Ewg, Hr3 and Blimp-1. Of these, both Hr3 and Blimp-1 are known direct targets of ecdysteroids (Akagi et al., 2016; Lam et al., 1999). Subsets of these four TFs form part of developmental timing mechanisms that control the expression of cell-surface molecules in neurons of the visual system (Jain et al., 2022) or neuronal pruning (Alyagor et al., 2018), providing additional support for our observation of co-operation between these factors and Ab to control adhesion. Significantly, though, while our functional studies show that all these TFs are necessary for dispersal of dorsal SHCs at pupariation, only Ab can trigger the complete loss of dorsal SHCs when expressed alone at wandering larval stages, indicating that Ab plays an instructive role.

We demonstrate that a key target of Ab in haemocytes are ninjurins, with repression of ninjurins by Ab enabling the developmental remodelling of SHCs in response to ecdysone (Fig. 7D,E). However, previous publications show that NijA expression and activity can be influenced by a number of additional stimuli. *NijA* transcription is responsive to immune triggers (Boutros et al., 2002; Buchon et al., 2009), while immune challenge can activate Mmp1 (Johansson et al., 2005), which, in turn, can cleave and regulate ninjurins (Jeon et al., 2020; Zhang et al., 2006), allowing haemocyte adhesion to be disrupted. In mammalian systems, LPS can bind human Ninj1 (Shin et al., 2016) and has the potential to disrupt ectodomain function and homophilic cell adhesion. We speculate that the regulation of Ninjurin expression and activity allows a wide range of stimuli to be integrated, enabling the release of haemocytes in response to a variety of signals. These include developmental signals such as ecdysone, as we have shown here (Fig. 7D,E), as well as immune challenges (Fig. 7F) and potentially even chemical signals such as oxygen tension (Shin et al., 2024). Such regulation by diverse inputs likely underlies the dispersal of SHCs that has been observed previously in response to a number of immune challenges and environmental stresses, and in melanotic tumour backgrounds (Honti et al., 2010; Letourneau et al., 2016; Markus et al., 2009; Stofanko et al., 2010).

While this work highlights the role of ninjurin-mediated homotypic interactions in maintaining SHCs, it is important to acknowledge that other factors, such as heterotypic interactions between the transmembrane protein Eater and collagen proteoglycans, have also been proposed to regulate SHCs (Csordás et al., 2020). Knockdown of either NijA or Eater results in loss of larval SHCs, suggesting that the final stereotyped pattern of SHCs may arise from an interplay between both heterophilic Eater-mediated matrix interactions, and homophilic NijA-mediated interactions between NijA-expressing haemocytes and spatially restricted non-haemocyte-expressed NijA.

The roles for ninjurins in haemocytes that we have demonstrated are entirely consistent with previous publications highlighting the immune functions of ninjurins in mammals. In mammals, ninjurins are expressed in myeloid cells and upregulated in endothelial cells under inflammatory conditions (Ifergan et al., 2011; Sorosina et al., 2022; Toma et al., 2020; Wang et al., 2017). Ninjurin knockout or antagonism has potent anti-inflammatory effects, indicating that the adhesive function of ninjurins is crucial for leukocyte transendothelial migration (Ahn et al., 2009; Jeon et al., 2020; Lee et al., 2018). This process, by which leukocytes move from the circulation to tissue, is essential for directing leukocytes to sites of activity and superficially resembles the recruitment we observe of haemocytes from circulating haemolymph into dorsal SHCs. A similar homing function for Ninj1 in mammalian leukocytes has been demonstrated in experimental autoimmune encephalomyelitis models, where increased Ninj1 expression in T cells in the lung facilitates their subsequent migration to the CNS (Odoardi et al., 2012). Although *Drosophila* lacks a vascular endothelium, these mammalian findings, together with our results, strongly support a conserved role for ninjurins in regulating the adhesion and homing of leukocytes to target tissues. It is tempting to speculate that SHCs could provide a simple, experimentally tractable system for identifying previously unreported conserved regulators of this fundamental process, which has profound importance for immune responses.

Finally, while our study has focused on the cell-adhesion functions of ninjurins, it is also important to consider that recent data implicate human Ninj1 in pyroptosis, agreeing with previous observations in *Drosophila* showing NijA is required for non-apoptotic cell death (Broderick et al., 2012). Pyroptosis is a form of cell death in which inflammasomes activate gasdermins to trigger oligomerization of Ninj1 and direct plasma membrane rupture (Kayagaki et al., 2021). The precise regulatory mechanisms by which Ninj1 is stimulated to oligomerize are unclear, but structural studies indicate that this is accompanied by the rearrangement of α-helices that are normally part of the extracellular domain. These α-helices insert into the plasma membrane, enable Ninj1 monomers to polymerize into amphipathic filaments (Degen et al., 2023) or rings, and allow disks of membrane to be excised (David et al., 2024), leading to the release of intracellular damage-associated molecular patterns that further drive inflammation.

Functional dissection of Ninj1 indicates that separate regions of the extracellular domain are required for the cell-adhesion or pyroptotic activities of Ninj1 (Araki and Milbrandt, 1996; Kayagaki et al., 2021). Specifically, an N-terminal region mediates adhesion, followed by an α-helical stretch that can insert into the cell membrane and facilitate Ninj1 oligomerization during pyroptosis. This raises the possibility that ninjurins are bifunctional immune regulators, having the ability to switch between mutually incompatible protein conformations that mediate either adhesion (exposed extracellular domain) or pyroptosis (membrane insertion). While our finding that ninjurins regulate dorsal SHC dynamics is consistent with this first adhesion function, pro-inflammatory pyroptotic activities for ninjurins in haemocytes remain to be established. An important objective going forward will be to identify the mechanisms that control ninjurin membrane conformation and the extent to which transitions in ninjurin membrane organization contribute to distinct haemocyte activities.

## MATERIALS AND METHODS

### Fly strains and genetic crosses

The following GAL4 drivers were used: *w^1118^*; *Pxn-GAL4, UAS-GFP* (Stramer et al., 2005) and *Hml-GAL4* (*w^1118^; P{Hml-GAL4.Δ}2* (Sinenko and Mathey-Prevot, 2004). UAS lines used were: *UAS-ab* (w*; P{UAS-ab.B}55), *UAS-NijA* (*P{EP}G4196*), *UAS-NijB* (*P{EPgy2}EY00277*), *UAS-Mmp1* (*P{UAS-Mmp1.f2}2*), *UAS-Mmp2* (*P{UAS-Mmp2.P}1*) and *UAS-Timp* (*P{UAS-Timp.P}1*). For crosses, 5-10 driver line virgin females were mated with 5 males of each UAS strain. All the crosses were maintained at 25°C, unless otherwise specified, and transferred to fresh vials every 2 days to prevent larval overcrowding and to enable sustained production of 3rd instar larvae from multiple vials over time.

Targeted ablation was by GAL4-inducible dsRNAi and shRNA. The *w^1118^, UAS-Dcr-2; Hml-GAL4, UAS-GFP* driver was generated by crossing *w^1118^; Hml-GAL4, UAS-GFP* with *w^1118^, P{UAS-Dcr-2.D}*; *+/+.* Virgin driver females were crossed with *UAS-RNAi* males, and crosses maintained at 25°C. The following dsRNAi lines were used: *ab^KK110195^*, *ab^GD4062^*, *NijA^KK113034^*, *NijA^GD2406^*, *NijB^KK111558^* and *NijB^GD16950^*. Temperature-sensitive *ecd^1^* mutants (Garen et al., 1977) were used to investigate ecdysteroid requirements for Ab expression.

Temporal control of GAL4-mediated Ab expression was carried out using the GAL80ts repressor. The *w^1118^; Pxn-GAL4, UAS-GFP; GAL80ts* driver was generated by crossing *w^1118^; Pxn-GAL4, UAS-GFP; +/+* with *w^1118^; +/+; GAL80ts (w; P{tubP-GAL80ts}2).* Virgin driver females were crossed with *UAS-ab* males and crosses were maintained at 18°C to repress GAL4. Progeny were shifted to 29°C to inactivate GAL80ts and induce *Pxn-GAL4*-mediated Ab expression.

Ecdysteroid signalling was visualized using the reporter strain *w^1118^; P{w[+mC]=EcRE.lacZ}SS4*, which contains seven copies of a 30 bp EcR response element unit from the *hsp27* promoter upstream of a minimal promoter element from the *Adh* gene and the coding sequence of β-galactosidase (Koelle et al., 1991). The temperature-sensitive allele *ecd^1^* was used to reduce ecdysteroid titres during third larval instar stages (Garen et al., 1977).

For analysis of SHCs, the following reporter strains were used: the larval muscle reporter *P{PTT-GB}Zasp52^G00189^*; the pericardial cell reporter *Dot-GAL4 (P{w[+mC]=Ugt36A1-GAL4.K}11C, P{w[+mC]=UAS-GFP.U}2*) and the haemocyte reporter *HmlΔ-DsRed.NLS* (Makhijani et al., 2011). NijA expression was revealed using the GFP gene trap y^1^ w*; Mi{MIC}NijA^MI15170^.

For analysis of transcription factor expression, the following GFP tagged lines were used: *w^1118^; Pbac{y[+mDint2] w[+mC]=Hr3-GFP.FLAG}VK00033*; *w^1118^; Pbac{y[+mDint2] w[+mC]=ewg-GFP.FPTB}VK00033*; y^1^ w*; P{y[+t7.7] w[+mC]=Blimp-1-GFP.FPTB}attP40; and y^1^ w*; Pbac{y[+mDint2] w[+mC]=gem-GFP.FPTB}VK00033/TM6C, Sb[1].

### Staging of 3rd instar larvae

For staging, larvae were raised on food supplemented with 0.05% Bromophenol Blue (Maroni and Stamey, 1983). By selecting larvae based on their foraging behaviour and colour of the gut, third instar larvae could be staged as: (1) ‘foraging’ stage larvae, which have blue guts and are continually feeding; (2) ‘wandering’ stage larvae, which have blue guts and move in an out of the media; and (3) ‘clear gut’ stage larvae, which have evacuated the larval gut and have left the medium. Analysing time to pupariation of these collection windows (Fig. S1) puts these stages at: (1) more than 30 h before pupariation; (2) 12-24 h before pupariation; and (3) 4-6 h before pupariation. Staging was performed by one investigator with collections transferred to numbered 35 mm Petri dishes for each genotype and stage, and then scored blind for dorsal SHCs by a second investigator.

### Scoring of dorsal sessile compartments and statistical analysis

Each experiment typically comprised multiple genotypes and stages alongside GAL4-driver crosses with control *w^1118^* flies, assayed side by side. The second investigator visualized and scored dorsal SHC number in each larva by manual inspection using an Olympus SZX12 stereo fluorescence microscope. Each larva was given a score between 0-6 corresponding to the number of dorsal SHCs present. The haemocyte GAL4 driver lines contain *UAS-*GFP transgenes that enable dorsal SHCs to be detected as GFP foci along the dorsal midline of larvae. Scored data from each collection were then collated and decoded by the first investigator to apply data to the relevant genotype and stage. A Kruskal–Wallis test, which is a non-parametric alternative to one-way ANOVA, was conducted to determine statistically significant differences in SHC numbers across genotypes and stages (wandering versus clear gut) for each experiment. Dunn's post-hoc tests were conducted to test pairwise comparisons and identify specific groups that differ significantly.

### Immunofluorescence microscopy of haemocyte populations

Circulating haemocytes were isolated from 10 staged wandering third instar larvae and processed for immunofluorescence microscopy using multispot slides (PH-001, C. A. Hendley), as described by Kwon et al. (2008). To prepare isolated SHCs for immunostaining, wandering third instar larvae were washed from medium under a stream of de-ionized water and dried, and dorsal cuticles were dissected in 200 µl of HyQ-CCM-3 culture medium (HyClone) containing protease inhibitors (Complete, Boehringer) while viewing using an Olympus SZX12 stereo GFP fluorescence microscope. Circulating blood cells were carefully washed away and the SHCs scraped off the cuticles using a tungsten needle into 50 µl culture medium on a multispot slide. Subsequent fixation and antibody incubation were as standard for circulating haemocytes.

Confocal microscopy was carried out using a Zeiss LSM 780 microscope. Antibodies used were: rabbit anti-Ab antibody (1:200, Hu et al., 1995), rabbit anti-beta-Galactosidase (1:5000; MP Biomedical, 559761), mouse Mab 40-1a anti-β-galactosidase (1:100; Developmental Studies Hybridoma Bank), rabbit anti-GFP (1:1000; Invitrogen, A11122). Secondary antibodies from Jackson Immunoresearch were Cy5-conjugated anti-mouse IgG (H+L) (715-175-151) and Cy3-conjugated anti-rabbit IgG (H+L) (711-175-152), all used at 1:400.

### Live imaging of haemocyte compartments

Wandering stage *HmlΔ-DsRed.NLS* (Makhijani et al., 2011) third instar larvae were staged as described above. For initial characterization of sessile compartment organization and haemocyte number, larvae were anaesthetized with Dichlorvos (Fluka, 1:1000 in 1XPBS), as described previously (Csordás et al., 2014). Anaesthetized larvae were immobilized via their ventral surface to insulin needles (Sterican 0.3×12 mm, B. Braun) using tissue adhesive (Liquiband Optima, OPT001). Larvae were imaged using a Zeiss Lightsheet Z.1 light-sheet fluorescence microscope in an imaging chamber containing 1×PBS with Dichlorvos. Image stacks were analysed in ImageJ (version 2.3.0/1.53q) using the TrackMate plug-in (Tinevez et al., 2017) to identify haemocyte nuclei and quantify nuclear number. For analysis of SHCs relative to other larval structures, *HmlΔ-DsRed.NLS* flies were crossed with either the larval muscle reporter *P{PTT-GB}Zasp52^G00189^* or the pericardial cell reporter *Dot-GAL4 (P{w[+mC]=Ugt36A1-GAL4.K}11C, P{w[+mC]=UAS-GFP.U}2*). Wandering stage larvae were staged as described above and larvae were anaesthetized by immersing in halocarbon oil (Oil 10S, Voltalef, VWR) containing 1:1000 Dichlorvos (Fluka). Individual larvae were mounted in an imaging chamber constructed by flanking larvae laterally with stacks of three borosilicate 22×22 No. 1 coverslips (MNJ-350-020H, Menzel-Gläser) overlaid with a borosilicate 22×50 No. 1 coverslip (MNJ-350-070P, Menzel-Gläser), and imaged immediately using a Zeiss LSM880 confocal microscope. Confocal *z*-stacks were analysed in ImageJ (version 2.3.0/1.53q).

### Haemocyte isolation

Primary haemocytes from third instar larvae were prepared as described previously (Kwon et al., 2016). Briefly, larvae were ripped in batches of 50 third instar larvae into HyQ-CCM3 insect medium (Thermo Fisher Scientific) containing protease inhibitors (Complete, Roche). For transcriptome analysis, cells were pelleted at 400* **g*** for 5 min, washed with ice-cold 1×PBS containing protease inhibitors and stored as pellets at −80°C until required. For chromatin immunoprecipitation sequencing (ChIP-seq), cells were fixed with 1% formaldehyde in 1×PBS for 15 min at 25°C and pelleted at 400* **g*** for 5 min then washed three times with ice-cold 1×PBS containing protease inhibitors and stored as pellets at −80°C until required.

### Real-time PCR analysis of transcript levels

For analysis of differential expression across larval stages, primary haemocytes from third instar *w^1118^* larvae were prepared as described above, with haemocytes corresponding to 300 animals used for mRNA isolation. For analysis of differential cell-adhesion gene expression, circulating haemocytes were bled from 300 wandering stage third instar *Pxn-GAL4, UAS-GFP; GAL80ts×UAS-ab* larvae that had been raised at 18°C. Circulating haemocytes were prepared as described above. To analyse cell-adhesion genes altered after *ab* ectopic expression, haemocytes were isolated 24 h after a temperature up-shift to 29°C from 600 larvae. mRNA was isolated from both populations using a μMACS mRNA Isolation kit (Miltenyi Biotec) according to the manufacturer's instructions. cDNA was generated by reverse transcription using Superscript II (Invitrogen) at 42°C. Real-time PCR was performed on an Applied Biosystems Step One Plus real-time PCR machine. Reactions were performed using Absolute QPCR SYBR green ROX mix (Thermo Fisher Scientific, AB-1162). Primers used are described in Tables S1 and S2. *RpL32* transcript provided the endogenous control for normalization.

### Abrupt ChIP-Seq

Haemocytes for chromatin immunoprecipitation-coupled sequencing (ChIP-Seq) were prepared as described previously (Kwon et al., 2008). Haemocytes were prepared from clear gut stage *w^1118^* larvae in batches of 50, as described above. ChIP was performed using Protein-A Dynabeads (Invitrogen) precoated with 2 µg of anti-Abrupt antibody (Hu et al., 1995). A total of 20 cell pellets (haemocytes from 1000 larvae) were resuspended in 100 µl of SDS Lysis Buffer (1% SDS, 10 mM EDTA) and chromatin sheared for 18 cycles in a Covaris S2 ultrasonicator using the following settings: 2 intensity, 200 cycles/burst, 10 s gap. Sheared chromatin was then diluted to 1 ml using ChIP dilution buffer (0.01% SDS, 1.1% Triton X-100, 1.2 mM EDTA, 16.7 mM Tris-Cl, 167 mM NaCl) and centrifuged at 21,000* ****g*** to separate the insoluble material and the soluble chromatin used for ChIP. Chromatin was pre-cleared using blocked, non-antibody coated Protein-A Dynabeads, 1% input sample reserved and ChIP performed by incubation for 2.5 h at room temperature with Anti-Ab coated Protein-A Dynabeads. Immune complexes were washed as described previously (Kwon et al., 2008), and protein-DNA complexes were eluted using two washes with 75 µl of elution buffer (1% SDS and 0.1 M NaHCO_3_ solution). ChIP DNA was liberated by addition of protease K (Roche) (20 µg) and incubation overnight at 65°C. DNA was purified using 1.8 volumes of AMPure XP beads (Beckman Coulter) and purified DNA eluted in 30 µl of 10 mM Tris (pH 8.0).

ChIP and Input DNA were sequenced using a SOLiD 4 genome analyser (Life Technologies). Barcoded fragment libraries for sequencing were generated using a DNA fragment library construction kit (Life Technologies) and multiplex barcoded adaptor kit (Life Technologies) according to the manufacturer's instructions. AMPure XP beads (as above) were used for all DNA clean-up steps. PCR amplification of libraries was monitored using 2.2% flash gels (Lonza) and amplification cycles stopped when fragment library bands were visible on flash gels. 17 cycles of amplification were used for ChIP and Input libraries. Libraries were sequenced using a SOLiD ToP Fragment Barcoded Sequencing Kit MM50/5 (Life Technologies), which generated 50 bp sequence reads from each DNA fragment. DNA sequence tags were mapped to the *Drosophila* genome (BDGP R5/dm3 Assembly) using the bioscope mapping tool (Life Technologies). Reads were then filtered for high-quality reads where read quality was greater than 15 using Samtools (Danecek et al., 2021). The numbers of mapped and filtered reads for ChIP and input libraries were 10,683,033 and 7,742,455, respectively. ChIP peaks were called using MACS (Zhang et al., 2008). MACS peak summits were used to identify flanking genes within 5 kb using a custom workflow in Galaxy (https://usegalaxy.org). Associated genes were imported into Cytoscape 3.2.0 and analysed using the iRegulon app (Janky et al., 2014) to identify regulatory networks that were then visualized in Cytoscape 3.8.2 using the yFiles circular layout option. GO network analysis was performed using the ClueGO Cytoscape plug-in (Bindea et al., 2009), as described previously (Kwon et al., 2020). The colour of nodes reflects significance of enrichment and size the number of genes. 250 bp DNA sequences flanking each Ab ChIP peak summit were extracted using the UCSC table browser tool. Motifs were detected using the Xstreme tool of the MEME motif discovery and enrichment analysis suite (5.4.1)

### mRNA-Seq

mRNA was extracted from haemocytes from 1000 larvae per library using a MACS mRNA Isolation kit (Miltenyi Biotec) and libraries prepared for sequencing using a SMARTer Stranded RNA-Seq kit (Clontech), according to the manufacturer's instructions, with the following modifications: heat fragmentation of RNA was performed for 3 min at 94°C. Libraries were barcoded and then run on an Agilent Bioanalyser 2100 with HS DNA chip to confirm library size centred on 300 bp. mRNA-seq libraries were sequenced using a commercial sequencing provider (Macrogen) on an Illumina HiSeq2500 sequencer. Read quality was assessed using FastQC (http://www.bioinformatics.babraham.ac.uk/projects/fastqc/). Reads were then mapped and expression determined using STAR (Dobin et al., 2013) and RSEM (Li and Dewey, 2011).

### CAGE-Seq

Total RNA was extracted from haemocytes from 1000 *w^1118^* larvae per library using an Rneasy Mini Kit (Qiagen) followed by DNase treatment, as described in the manufacturer's instructions. CAGE-Seq libraries were prepared and sequenced by DNAForm (Riken, Japan) and mapped to the genome using bwa (Li and Durbin, 2009). Reads were used to determine TSS locations genome-wide in wild-type haemocytes. Specifically, BED file containing all CAGE reads for each genotype was used to determine read number at each genome co-ordinate using the genomecov utility of bedtools (Quinlan and Hall, 2010). Tracks were filtered to define bona-fide initiation points in wild-type by filtering for genome intervals containing more than 10 mapped reads. Peak value, which defines the TSS in each interval, was then determined using the bigWigSummary utility of kentUtils (https://github.com/ENCODE-DCC/kentUtils). For analysis of *Drosophila* TFs, CG numbers for all FlyBase-annotated TFs were obtained from https://flybase.org/reports/FBgg0000745.html#synonyms and used to define genomic co-ordinates of TFs. These intervals were used to extract normalized CAGE read number for each fly TF.

### Protein sequence analysis

Membrane topology predictions for *Drosophila* ninjurins were generated using the TMHMM server (http://www.cbs.dtu.dk/services/TMHMM/), while prediction of intrinsically disordered regions for fly and human ninjurins were made using PONDR (Peng et al., 2006) and PrDOS (https://prdos.hgc.jp). Matrix metalloprotease cleavage sites were predicted using DeepCleave (Li et al., 2020). Secondary structure predictions were made using PSIPRED (http://bioinf.cs.ucl.ac.uk).

### Ecdysteroid regulation of Ab expression

Haemocytes were isolated from homozygous mutant *ecd^1^* larvae raised at a permissive temperature of 22°C and then shifted to the restrictive temperature of 29°C after the 2nd larval instar to inactivate Ecd. Haemocytes were also isolated from foraging stage (non-Ab expressing) and clear gut stage (Ab-expressing) *w^1118^* larvae. Haemocytes were isolated from 20 larvae of each genotype and processed for anti-Ab immunofluorescence microscopy using multispot slides, as described above.

### Survival and infection assays

Survival in response to septic wounding with *Enterococcus faecalis* (a gift from B. Lemaitre, EPFL, Switzerland) and *Pseudomonas entomophila* (a gift from H. Williams, Imperial College London, UK) was tested. *E. faecalis* was maintained on LB medium at 37°C. Overnight cultures were seeded into 200 ml LB in a 1 l conical flask and incubated overnight at 37°C in an orbital shaker with minimal shaking (max rpm 20), pelleted and resuspended to OD_600_∼200 in 1× PBS. *P. entomophila* was selected on LB agar plates containing 10% non-fat milk powder (Marvel) at 30°C. Colonies with a clear halo were seeded into 3 ml LB starter cultures at 30°C in a shaking incubator. Starter cultures were added to a final volume of 200 ml LB in a 1 l conical flask, incubated overnight at 30°C in an orbital shaker with shaking (rpm 250), pelleted and resuspended to OD_600_∼200 in 1× PBS. Survival was assayed in *w^1118^*; *Pxn-GAL4, UAS-GFP/+* and in control (*w^1118^/UAS-NijA*) and NijA overexpressing (*w^1118^*; *Pxn-GAL4, UAS-GFP/UAS-NijA*) 3rd instar larvae. For each larval strain, five replicates of 30-40 larvae were harvested and transferred to 35 mm diameter apple juice agar plates [50% pure clear apple juice, 2% BD Bacto Agar (Fisher Scientific), 0.5% methyl 4-hydroxybenzoate (Merck) and 0.8% propionic acid (Merck)]. Larvae were wounded ventrally between the A7 and A8 abdominal segments using a fine sterilized tungsten probe (0.6 µM tip, 25 mm length, Agar Scientific) that had been dipped in either sterile 1× PBS or the respective bacterial slurry. The needle was further sharpened on a diamond sharpening card while viewing using a stereomicroscope, and sterilized in ethanol with flaming before each wounding experiment. After infection, plates were incubated at 30°C, and survival recorded every 2 h for 10 h. Kaplan–Meier survival analysis was performed using SPSS to estimate the survival function for all populations. Pairwise comparisons Log Rank (Mantel-Cox) were conducted to evaluate the differences in survival between populations.

## Supplementary Material



10.1242/develop.202977_sup1 Supplementary information

Table S1. Primer sequences for real-time PCR analysis of adhesion molecules.
