# Supplementary material for: Abrupt-mediated control of ninjurins regulates Drosophila sessile haemocyte compartments
Source: Development. 2024 Dec 9;151(23):dev202977. doi: 10.1242/dev.202977 (PMC11664169; doi:10.1242/dev.202977)
Supplement: Supplementary information [file develop-151-202977-s1.pdf]

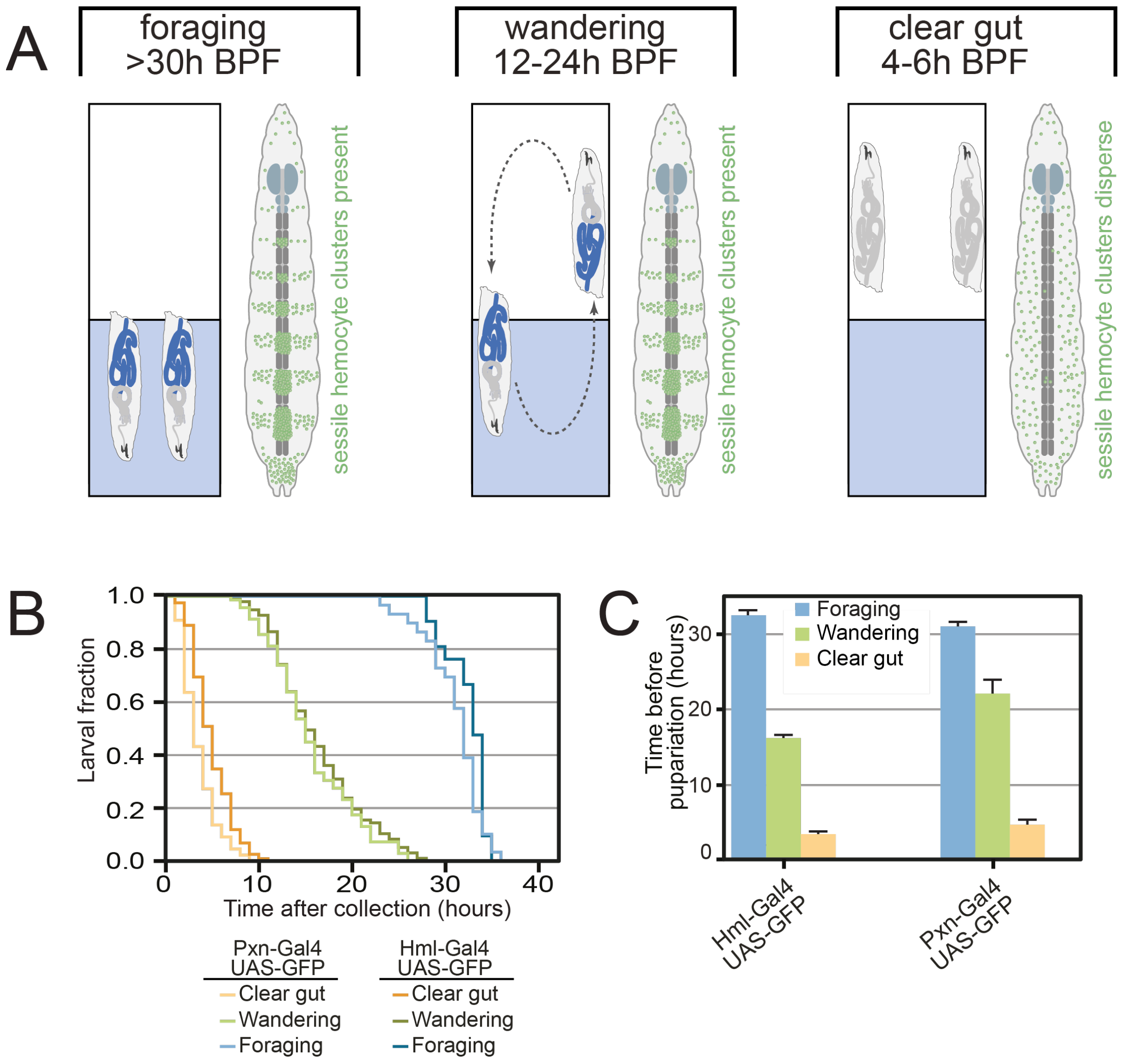

Fig. S1. Validation of larval staging methods and age classes. (A) Third instar larvae can be staged and sorted into three age classes - “foraging”, “wandering” or “clear gut” - based on gut contents and foraging/wandering behaviour. Foraging larvae are present 30h before pupa formation (BPF), are actively feeding and adopt a characteristic head-down feeding position with the only the posterior spiracles out of the media. Wandering larvae are present between12-24h BPF and exhibit a behavioural switch where larvae begin to repeatedly emerge from and return to the media, behaviour termed either wandering or roving. At both foraging and wandering stages, the gut contains food that can be visualized if dye has been added to the media. In this case bromophenol blue has been added which gives a blue-gut phenotype. Clear gut larvae present 4-6h BPF, exhibit additional changes in behaviour where they cease wandering, remain out of the media and purge gut contents resulting in the loss of food dye and a clear gut phenotype. Enlarged larval diagrams at each

Development • Supplementary information

stage indicate organisation of sessile hemocyte compartments, that can be detected at foraging and wandering stages but are dispersed at clear gut stages. (B-C) To confirm timings of the "foraging", "wandering" or "clear gut" stages relative to pupariation, third instar larvae were sorted into these categories based on gut contents and foraging/wandering behaviour and then monitored hourly after collection to determine time after collection at which individual larvae pupariated. Pupariation was scored based on eversion of anterior spiracles. Times for each larva to pupariation were recorded as events. By treating pupariation as an event like death, survival analysis methods based on the Kaplan-Meier estimator could be used to calculate the cumulative probability at hourly time points after collection that individuals in a sample were larval or pupal. (B) Modified Kaplan-Meier survival plot showing cumulative larval probability for staged populations of *Pxn-GAL4, UAS-GFP/+* and *Hml-GAL4, UAS-GFP/+* larvae 3rd instar larvae. (C) Mean time to pupariation for staged populations of each genotype.

Development • Supplementary information

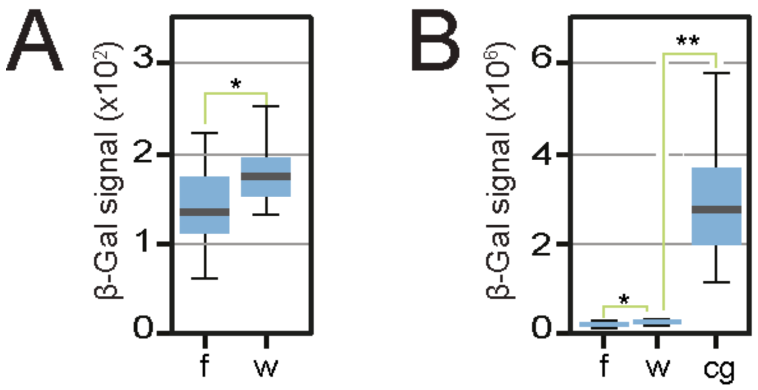

Fig. S2. EcRE-lacZ activation in third instar larval hemocytes.

P{w[+mC]=EcRE.lacZ}SS4 Larvae were collected, staged, and sorted into foraging (f), wandering (w) or clear gut (cg) classes as described in Fig. S1. Hemocytes were isolated and immunostained using anti-lacZ antibodies to reveal reporter activation and signal intensities analysed following confocal microscopy (A) Comparison of signal intensities in foraging (f) and wandering (w) stage larvae indicates statistically significant increase in reporter activation in wandering larvae. (B) Comparison of signal intensities in all larval populations shows significant activation of EcRE-lacZ in clear gut stage larvae. * indicates p-value of 0.0001; ** indicates p-value of 4.5E-18.

Development • Supplementary information

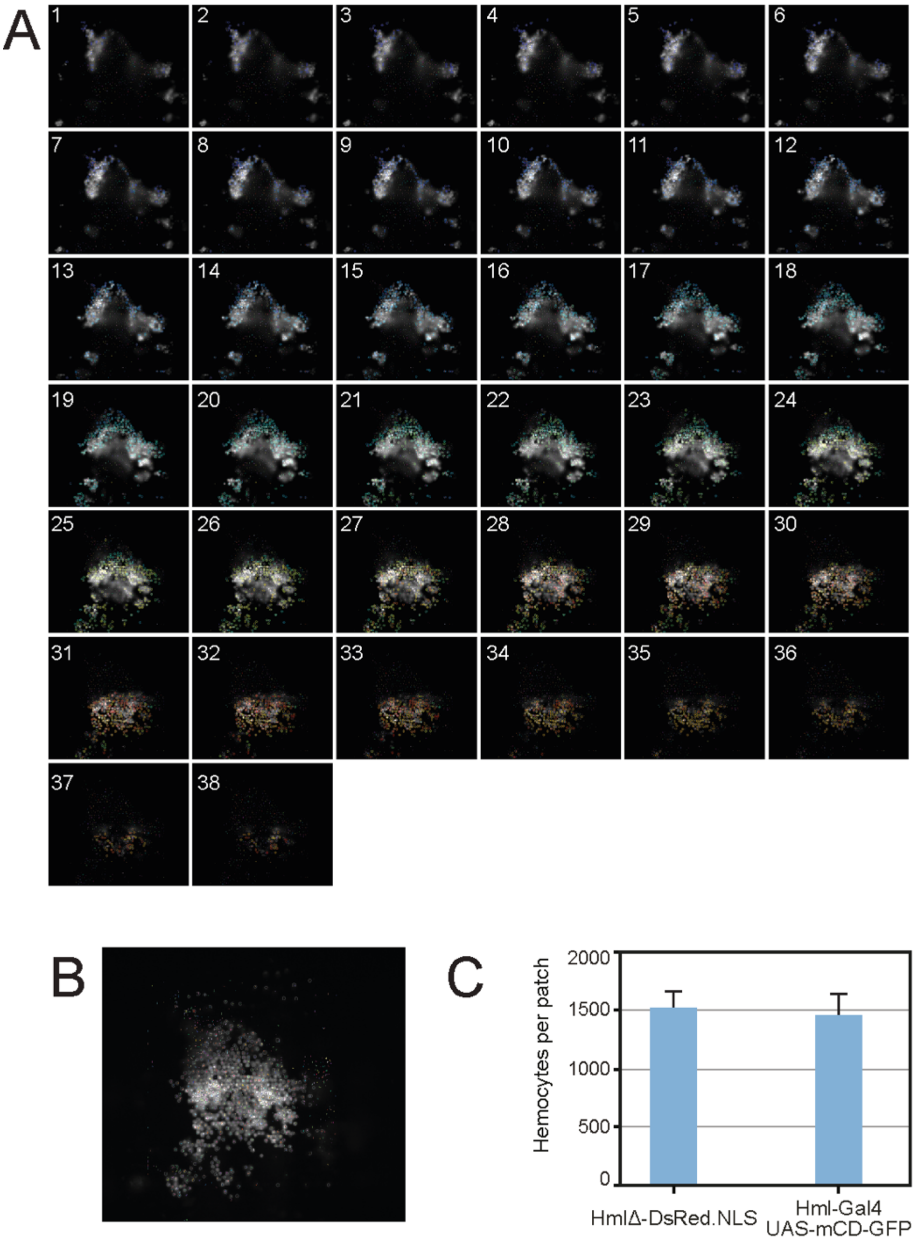

Fig. S3. Live imaging and cell tracking of dorsal sessile compartments. (A) Montage of Z-stack images through posterior dorsal sessile compartment of *HmlΔ-DsRed.NLS* third instar larva. In each section nuclei were identified using spot-finding tool of the ImageJ *TrackMate* plugin (coloured circles) and nuclei tracked through each Z-stack using the simple LAP tracker of *TrackMate* to identify tracks corresponding to individual cells. The total number of such "tracks" identified corresponded to total nuclei and thus cell number. Similar methods were used for *Hml-GAL4, UAS-mCD8-GFP* larvae that express GFP in hemocyte cell membranes. (B) High magnification image of a single slice from the Z-stack in (A) showing tracked-nuclei. (C) Graph showing average of hemocyte numbers in posterior dorsal sessile compartments of *HmlΔ-DsRed.NLS* and *Hml-GAL4, UAS-mCD8-GFP* third instar larvae. Data are mean and standard deviation of 10 independent larvae for each genotype.

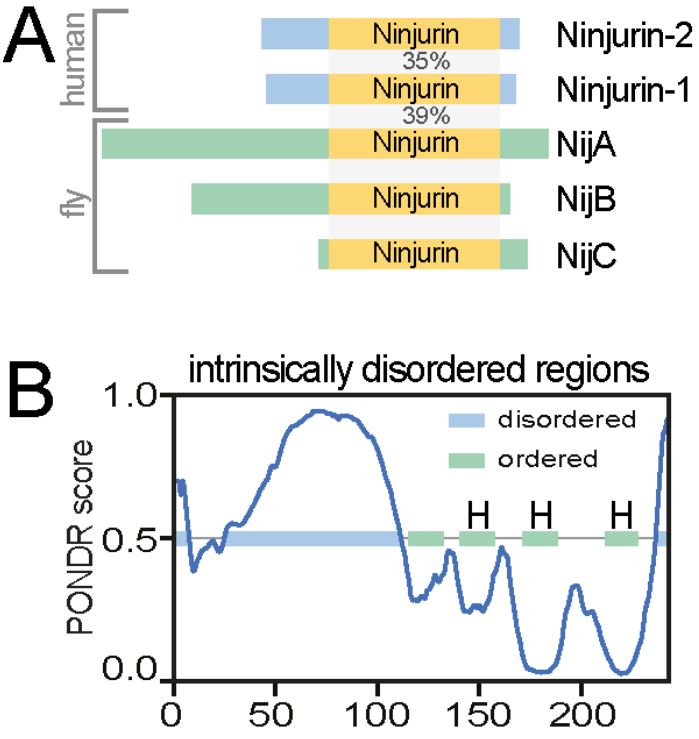

Fig. S4. Conservation and domain organisation of Ninjurins. (A) NijA and its paralogues NijB and NijC share sequence conservation with mammalian Ninjurin 1 and Ninjurin 2, all possessing a conserved Ninjurin domain. (B) Analysis of protein disorder indicate Ninjurins adopt a distinct conformation with a variable N-terminal extracellular domain (ECD), a core transmembrane (TM) domain with short, C-terminal intracellular domain (ICD). H in (B) indicates predicted helices (PSIPRED). High PONDR score indicates ECD contains regions with high propensity to form intrinsically disordered domains.

Table S1. Primer sequences for real-time PCR analysis of adhesion molecules.

Available for download at
https://journals.biologists.com/dev/article-lookup/doi/10.1242/dev.202977#supplementary-data

Development • Supplementary information

Table S2: Primer sequences for real-time PCR and semi-quantitative PCR analysis.

Gene	Name of Primer	Primer Sequence (5'-3')
NijA	NijA-F	TGTTGTGAGCTCGTCTCCAG
	NijA-R	GAGACACCCATACCCGATGT
NijB	NijB_F	CGCCAGTACAACCAGTGGAC
	NijB_R	CTGGATGGGAACTGGTCGTC
NijC	NijC_F	GGATGCCAATAGGTATGCC
	NijC_R	CCTGGAGGATGTACTTCAGCT
ab	ab_F	CACAGCCTTCCAGTTCCAAG
	ab_R	GCAGGACCAGTACAACTCA
Blimp-1	Blimp-1_F	GACCACCGCCAGCCAGTTGC
	Blimp-1_R	GCTGCTGCTGTTGCTGTTGC
Hr3	Hr3_F	GAACGCAATGGAGTGCGTGG
	Hr3_R	CAGCAGTGTGTCCAGCACGG
gem	gem_F	GCAGCGGCTCCAATATCTTC
	gem_R	GGATCACCTTGCCGGCCTGC
ewg	ewg_F	GGCATCAGGTCACTCCTGGC
	ewg_R	GAGTGGCCAAGACTATGGGC
rp49	rp49_F	CAACAGAGTGCGTCGCCGCTTCAAGGGACA
	rp49_R	CAGCTCGCGCACGTTGTGCACCAGGAACTT

Development • Supplementary information
