# Supplementary material for: Abrupt-mediated control of ninjurins regulates Drosophila sessile haemocyte compartments
Source: Development. 2024 Dec 9;151(23):dev202977. doi: 10.1242/dev.202977 (PMC11664169; doi:10.1242/dev.202977)
Supplement: Supplementary file 1 [file develop-151-202977_review_history.pdf]

Abrupt-mediated control of ninjurins regulates *Drosophila* sessile haemocyte compartments

So Yeon Kwon, Kimberley Chan, Martin Stofanko, Ka Hei Chan and Paul Badenhorst
DOI: 10.1242/dev.202977

Editor: Thomas Lecuit

Review timeline
Original Submission: 30 September 2021
Editorial Decision: 26 October 2021
Resubmission: 11 June 2024
Editorial Decision: 16 August 2024
First Revision Received: 2 October 2024
Accepted: 31 October 2024

Original submission

Original decision letter

MS ID#: DEVELOP/2021/200209

MS TITLE: Abrupt-mediated control of Ninjurin cell adhesion molecules regulates Drosophila sessile hemocyte compartments

AUTHORS: So Yeon Kwon, Kimberley Chan, Martin Stofanko, and Paul Badenhorst

I have now received all the referees' reports on the above manuscript, and have reached a decision. The referees' comments are appended below, or you can access them online: please go to BenchPress and click on the 'Manuscripts with Decisions' queue in the Author Area.

As you will see, the referees express considerable interest in your work, but have some significant criticisms and recommend a substantial revision of your manuscript before we can consider publication. If you are able to revise the manuscript along the lines suggested, which may involve further experiments, I will be happy receive a revised version of the manuscript. Your revised paper will be re-reviewed by one or more of the original referees, and acceptance of your manuscript will depend on your addressing satisfactorily the reviewers' major concerns. Please also note that Development will normally permit only one round of major revision.

We are aware that you may be experiencing disruption to the normal running of your lab that make experimental revisions challenging. If it would be helpful, we encourage you to contact us to discuss your revision in greater detail. Please send us a point-by-point response indicating where you are able to address concerns raised (either experimentally or by changes to the text) and where you will not be able to do so within the normal timeframe of a revision. We will then provide further guidance. Please also note that we are happy to extend revision timeframes as necessary.

Reviewer 1 Advance Summary and Potential Significance to Field:
The authors investigate the role of Abupt in controlling hemocyte adhesion to sessile patches in 3rd instar larvae. Through an analysis of lateral sessile compartments in larvae they use gain and loss of function experiments to show that Abrupt controls ninjurin cell adhesion molecules, which through some unknown mechanism regulates sessile compartments. This is interesting as ninjurins have primarily been characterised in the nervous system. Hemocytes and cells of the nervous system are

© 2024. Published by The Company of Biologists under the terms of the Creative Commons Attribution License (https://creativecommons.org/licenses/by/4.0/).

known to share common developmental programs (e.g. the GCM transcription factor) and this new work suggests the existence of additional shared regulatory mechanisms.

Reviewer 1 Comments for the Author:
While this work is intriguing the manuscript suffers from overspeculation and a lack of focus. The authors perform mRNAseq, CAGEseq (they never explain what this is), and Chipseq, and it is unclear how all of these trendy techniques add to the story. mRNAseq could probably have been sufficient. Instead they use all of these sequencing data to try and figure out a larger network of transcription factors that regulates Abrupt without any experiments to back this up. This is far too speculative and it is difficult to interpret these results.
Indeed, the authors interpret their CAGE-seq and RNA-seq experiments by stating that these experiments are "'confirming potential' roles in a broader regulatory network". This seems to be an oxymoron. Either experiments 'confirm' or highlight 'potential' functions. Similarly, their analysis of ninjurin domain structure is beyond the scope of the manuscript. While they use this data to discuss the interesting possibility of ninjurins in liquid-liquid phase separation, this is again far too speculative.

In my opinion the authors should decide on a single message and focus the manuscript (possibly shortening the paper into a shorter format). There are still open questions regarding the sessile patches that the authors could address.

Specific comments:

1)Most of their analysis of sessile compartment is based on stratifying the 3rd instar larval ages through their feeding behaviour. This is an approach that I am not familiar with. I noticed that in the methods they reference some older papers. Is this a standard approach? Could the authors do a timecourse to confirm that they are indeed separating the stages correctly? What if a perturbation affects feeding behaviour?
2)The authors focus on lateral sessile patches. But what about the dorsal patches? Are these not regulated by Abrupt?
3)When they overexpress abrupt they show that hemocytes get relocalised to the dorsal vessel (Fig. 3). Interesting, but do they know why?
4)The authors do not quantify the sessile patches in this experiment in a similar way to other experiments (e.g. in Fig. 5 they actually count sessile patches). Additionally, they do not examine dorsal vessel targeting in any other experiments. This is to say that their choice of analysis seems inconsistent.
5)The authors mention a few times that MMPs regulate ninjurins. Do they know if MMPs are also involved in controlling these sessile patches?

Minor comment-
1)Please add labels showing Figure numbers.
2)It is not good practice to use red/green overlays (https://www.ascb.org/science-news/how-to-make-scientific-figures-accessible-to-readers-with-color-blindness/).

Reviewer 2 Advance Summary and Potential Significance to Field:
In this interesting report, Kwon and colleagues investigate a mechanism that leads to the dispersal of Drosophila immune cells from large clusters on the dorsal surface of the developing larvae. This coincides with a peak in the steroid hormone ecdysone and changes in larval behaviour. Based on previous work they chose to investigate the role of a transcription factor called Abrupt. Abrupt levels correlate with ecdysone signalling in the insect blood cells (hemocytes) and in the absence of Abrupt the dispersal process is disrupted. Screening of potential mediators of cell adhesion led the authors to investigate whether the Ninjurin family of homophilic cell adhesion molecules were responsible for maintaining clusters and were able to show downregulation of these genes at the correct point in development, while their overexpression strengthens cluster formation and hinders dispersal. Knocking Ninjurins down leads to precocious dispersal, suggesting these play a role in cluster formation and antagonism of their expression facilitates release.

A variety of molecular and bioinformatic approaches were then used to investigate a potential transcription factor network in remodelling of hemocyte function and identify potential DNA motifs, though the importance of these other factors was not addressed, nor the potential of these

© 2024. Published by The Company of Biologists under the terms of the Creative Commons Attribution License (https://creativecommons.org/licenses/by/4.0/).

motifs as binding sites investigated experimentally beyond the bioinformatics. In summary, the authors present an interesting model whereby Ecdysone triggers Abrupt expression and leads to repression of Ninjurin to facilitate dispersal of hemocyte clusters.

This is an interesting story and largely the data is robust and well presented and of interest to the field. However, I feel the importance and relevance of this mechanism could be developed a little and further investigation of some key points may make the story more suitable for publication in Development. The clusters that this work investigates have been described by several groups but their significance and importance remains unclear. The work has potential to shed light on this but could reasonably easily be expanded to increase its impact or validate the role of novel molecular players (new transcription factors and motifs identified towards the end of the paper) beyond Abrupt that was itself identified through an earlier study. There are some minor points that need to be addressed to help make the conclusions more substantiated/robust.

Reviewer 2 Comments for the Author:
Major points

Authors suggest understanding the mechanism behind homing to sessile clusters will enable dissection of their role. This is easy to do now they have uncovered the mechanism – can they show some of the consequences of its manipulation? What are the point of these clusters – is proliferation affected if this mechanism is perturbed? In the discussion authors suggest other situations in which clusters may disperse – can they look at one or more of these to address how critical/relevant this mechanism is? (immune challenge, environmental stress, tumours). Addressing the importance and relevance of this mechanism would go a long way to strengthening the paper and broaden the range of interest in this work.

Does this mechanism influence hemocyte biology at other ecdysone peaks? It looks there is low level of Abrupt expression in hemocytes – could this relate to previous peaks via protein perdurance?

Does interfering with Ecdysone signalling block hemocyte relocalisations in vivo? If already known these papers should be cited, if not, this should be done. Given the role of Ab in other cell types can authors show that Ab expression is affected by EcR signalling in a hemocyte-autonomous manner (e.g. using Dominant negative or active versions of EcR expressed specifically in hemocytes)? On page 6 authors may wish to cite references that back up correlation of ecdysone signalling levels with the presence of dye in the larval gut/changes in larval behaviour (I realise these are in the methods). Why is the activation of the LacZ reporter so heterogeneous in the larval hemocyte population? Can authors show in vivo staining to demonstrate that it is the LacZ positive cells (or a live reporter) specifically leave clusters.

Where else are the Ninjurin’s expressed? The model in Figure 7 suggests hemocytes are using Ninjurin’s to attach to tissue as well as each other. This mechanism may account for hc-hc adhesion but do Ninjurin’s expressed elsewhere help this process? If it is just hc-hc adhesion this does not explain localisation in these specific clusters. High resolution (in z)/live imaging of some of these clusters as they disperse may help develop the model further, though I recognise those would be technically challenging experiments.

Relocalisation is not quantified in the early figures, though comparable genotypes are quantified in Figure 5. Where have the hemocytes gone in Fig 3D - can we be sure the hemocytes have not largely been killed off due to this misexpression strategy? Whole larvae should be shown alongside quantification of total hemocyte numbers here. Why does Figure panel 3D not look more like 3C if the cells are dispersed? Why do we see this weird localisation at the dorsal vessel rather dispersing as per 3C? Total numbers of cells is important as fewer hemocytes will mean fewer available to stick in these clusters which will in turn make them less obvious (and may also provide insight into importance with respect to haematopoiesis). Scoring of clusters is not explained in detail and may be subjective. How are dorsal patches defined (how big do they need to be to be scored?) I can’t see how this was done in the methods. Was the scoring of what appears to be a slightly subjective phenotype done on blinded samples?

© 2024. Published by The Company of Biologists under the terms of the Creative Commons Attribution License (https://creativecommons.org/licenses/by/4.0/).

The report identifies a number of transcription factors and motifs potentially involved but their importance is not addressed experimentally. Can the role of one or more of these be investigated with respect to relocalisation of hemocytes and so test/support the bioinformatic/molecular profiling data? To address the importance of the motifs likely requires enhancer analysis/new transgenics and is probably beyond the scope of this report (unless the authors get very lucky with publicly available mutants) so the role of the transcription factors seems easiest to address in their imaging of clusters assay. I think this would add to the story and certainly round it off nicely.

Minor points

Authors claim no expression of LacZ in Fig1 D,G but green channel fluorescence clearly visible. This requires some sort of negative control to make this statement as this data could also be interpreted as low/high levels of LacZ. Also this is not uniform across all hemocytes so some quantification of proportions would be helpful.

Fig. 2 – again some Abrupt staining appears present in absence of high levels of LacZ. Correlations between Abrupt and LacZ should be quantified in some way for reproducibility. Given Fig 2L's result authors may want to consider perdurance of protein from previous bouts of ecdysone signalling. At the end of page 7 it is stated that Ab is absent; this should be rephrased as low levels in comparison to when ecdysone signalling is intact.

I cannot find Figure S1 is cited in the text. Why are different regions of the embryo used to show absence of/expression of Abrupt in Fig S1 E and F? The same region of the embryo should be used.

Figure S2 – I think there is GFP expression when Gal80[ts] flies are raised at 18oC. Though it is definitely reduced (the presence of GFP would be more obvious if a more restricted band pass filter had been used to image larvae). Authors should probably modify statements as it looks like (according to GFP's presence) that low levels of Ab may be present despite the Gal80[ts] strategy at 18oC.

Some of the references are not formatted correctly.

Fig 2K cited ahead of G-J – move panel over to the left so K becomes 2G to help reader?

P8 2nd sentence – missing "we" from start of sentence

Figure 1A and Figure 3A look to be the same image. Can authors use different images?

The 3D structures are beautiful and interesting but it's not clear how this information progresses the main story here. Perhaps this could re-written a little to this data in to the overarching thread more clearly as, currently, it feels like a slightly superfluous (but pretty) addition.

The analysis of bled hemocytes looks robust, but can authors confirm how well their methods release cells attached to the body wall as this may influence magnitude of the effects they see?

Image in 5B is not as convincing as in other figure panels for the equivalent control.

Page 11 "snap-back darns" – "RNAs"?

Panel 6E needs more explanation in the text/legend as for those not familiar with iRegulon this is very hard to interpret.

Figure S3 – no legend. Can some non-expressed TFs be shown as a comparison?

Check all supplementary tables and figures are cited in the text. I think some are missing.

© 2024. Published by The Company of Biologists under the terms of the Creative Commons Attribution License (https://creativecommons.org/licenses/by/4.0/).

Author response to reviewers' comments

Response to reviewers
We would like to express our gratitude to the reviewers for both their positive and their critical comments. We have addressed all of their concerns and feel that the manuscript has improved considerably as a result. Most importantly, we have now performed additional experiments and made text changes that we feel help to focus the manuscript and provide further supporting evidence for our model.

Below, please, find a point-by-point reply to the reviewer's comments (reviewer comments in italics). We have taken the liberty of numbering each of the reviewers comments to allow easier cross-referencing between responses.

Reviewer 1 Comments for the Author:
(1) While this work is intriguing the manuscript suffers from overspeculation and a lack of focus. The authors perform mRNAseq, CAGEseq (they never explain what this is), and Chipseq, and it is unclear how all of these trendy techniques add to the story. mRNAseq could probably have been sufficient. Instead they use all of these sequencing data to try and figure out a larger network of transcription factors that regulates Abrupt without any experiments to back this up. This is far too speculative and it is difficult to interpret these results.
Indeed, we agree with the reviewer that this section of the work in the submitted manuscript was too speculative and superficial.

We have strengthened this section by performing additional experiments that include:

- *i) Comparison of expression of these transcription factors to known hemocyte regulators to confirm that their expression is at the level of other factors with demonstrated function (new Fig. 6B)*
- *ii) Analysis of expression profile of these TFs across the developmental time course from feeding, wandering to clear gut larval stages confirming that expression of these factors mirrors expression of Ab and expression increases occur when hemocytes are dispersed from sessile compartments (new Fig. 6C)*
- *iii) Use of GFP fusions with these TFs to confirm that they are translated and nuclear-localised in hemocytes when sessile compartments disperse (new Fig. 6D)*
- *iv) Performed hemocyte-specific over-pression and also RNAi to demonstrate that all TFs are required for hemocyte dispersal from sessile compartments (new Fig. 6E), confirming function. Over-expression demonstrates that unlike Ab which is sufficient to cause early hemocyte dispersal none of these TFs can trigger complete dispersal when over-expressed alone (new Fig. 6E).*

We trust these additional work helps to validate this network of factors as not only being expressed but also being functionally required for this process.

We have retained both the CAGE-seq and mRNA-seq data as we feel they provide complimentary information sets when interrogating expression of targets. CAGE-seq is by no means an especially trendy technique, having been first developed 18 years ago, but does provide invaluable information on transcription start sites (TSSs) and enables accurate quantitation of transcript abundance. Because CAGE sequencing libraries focus specifically on TSSs and do not cover other elements of transcripts, they allow high read coverage to obtained for just TSSs regions of the transcript without having to perform large amounts of sequencing. This makes CAGE-seq cost-effective while the high read coverage enables accurate quantitation of transcript abundance. In contrast, mRNA-seq provides information across the transcript that enables alternative splice junctions and isoforms to be identified. However, robust calling of expression levels by mRNA-seq, especially for low abundance transcripts, requires significantly greater sequencing levels to obtain the required coverage across the entire transcript. As such CAGE-seq and mRNA-seq provide complimentary information that together provides the best measure of transcript abundance as well as organisation. We have therefore kept both sets of data in the paper but have added a brief description to the text that makes clear the rationale for choosing both of these methods to analyse transcription. Text added is:

© 2024. Published by The Company of Biologists under the terms of the Creative Commons Attribution License (https://creativecommons.org/licenses/by/4.0/).

"Expression was monitored both by CAGE-seq (Cap analysis of gene expression sequencing), to discriminate and monitor the activity of transcription start sites (TSSs), as well as by mRNA-seq to determine splice variants."

(2) Indeed, the authors interpret their CAGE-seq and RNA-seq experiments by stating that these experiments are "'confirming potential' roles in a broader regulatory network". This seems to be an oxymoron. Either experiments 'confirm' or highlight 'potential' functions.
Indeed, this has been removed. In addition to being an oxymoron, this speculative phrase is now redundant as we have actually *demonstrated requirements for these factors by a new set of hemocyte-specific RNAi and over-expression studies (new Fig. 6E).*

(3) Similarly, their analysis of ninjurin domain structure is beyond the scope of the manuscript. While they use this data to discuss the interesting possibility of ninjurins in liquid-liquid phase separation, this is again far too speculative.
We agree with this reviewer and have now removed these sections as they are overly speculative. While we still believe that liquid-liquid phase separation contributes to Ninjurin adhesive functions, this discussion is best left to future manuscripts that specifically test this activity and is beyond the scope of this manuscript. Thank you for pointing this out.

(4) In my opinion the authors should decide on a single message and focus the manuscript (possibly shortening the paper into a shorter format). There are still open questions regarding the sessile patches that the authors could address.
We agree with this reviewer and have attempted to focus the manuscript on sessile patches and their regulation. We have not shortened the manuscript as we have added substantial additional work but have removed sections that did not contribute to the principal message of the paper – the role of Ninjurins in the maintenance of sessile hemocyte compartments and how expression of Ninjurins is controlled to effect release of hemocytes at pupariation.

(5) Most of their analysis of sessile compartment is based on stratifying the 3rd instar larval ages through their feeding behaviour. This is an approach that I am not familiar with. I noticed that in the methods they reference some older papers. Is this a standard approach? Could the authors do a timecourse to confirm that they are indeed separating the stages correctly? What if a perturbation affects feeding behaviour?
This is an approach that has been around for a number of years and was used extensively in the older papers, specifically time courses of expression of ecdysone-inducible genes from mid 3rd instar larval stage through to pupal stages. It enabled some quite defined stage collections of larvae to be made which allowed distinct sets of ecdysone responders to be discriminated. The alternative approach for staging is to make very short egg collections and then age the resultant eggs and resultant larvae for up to 120 hr after egg laying (AEL). However, while this approach can be reasonably reliable when using a single strain under the right conditions, there can be a high dispersion and loss of synchrony between individuals of the population over this 5-day time period. This is especially so if larvae are crowded and if strain backgrounds differ. The blue-gut method allows staging closer to the point of assay which limits this loss of synchrony in the collection.

The reviewer made an excellent suggestion whether we could confirm that we are indeed staging correctly by performing a timecourse after staging to confirm that we have staged correctly. This was a great experiment which we have now performed and added as *new data in Supplementary Figure S1A and S1B. Specifically, we staged larvae from both Hml-GAL4, UAS-GFP and Pxn-GAL4, UAS-GFP driver crosses with w[1118]. These crosses were selected as they are the driver crosses used for all our hemocyte over-expression and RNAi experiments. Larvae were staged into the 3 classes using our staging methods: foraging, wandering, clear-gut stages and then placed on apple-agar plates and monitored hourly to score time after collection when larvae pupariated (pupariation was scored based on eversion of anterior spiracles). We treated pupariation times for each larva as a "event" and used Kaplan-Meier curves to analyze time-to-event data for multiple independent collections. In our experiments the event recorded is pupariation. This allowed us to determine i) times-to-pupariation for each collection method and ii) show that there was little difference between strains for calculated times-to-pupariation for each collection.*

While this data is added as supplemental material, we have included the following text to the main text of the paper that summarises and refers to these results. Specifically, "As shown in Fig.

© 2024. Published by The Company of Biologists under the terms of the Creative Commons Attribution License (https://creativecommons.org/licenses/by/4.0/).

S1, by monitoring the switch from foraging to wandering behaviour, and by visualising gut contents to determine gut purging, we were able to stage and segregate third instar larvae into three distinct age classes relative to pupariation. These we have termed respectively: i) foraging larvae that are >30 hours before pupariation with no ecdysteroid signalling, ii) wandering larvae that are 12-24 hr before pupariation and which experience the small rise in ecdysteroid levels that initiates wandering behaviour, and iii) clear gut larvae which are 4-6 hr before pupariation, and which experience the high titre ecdysteroid pulse that triggers the onset of pupariation."

(6) The authors focus on lateral sessile patches. But what about the dorsal patches? Are these not regulated by Abrupt?

The dorsal sessile patches are indeed regulated by Abrupt. The bulk of the manuscript does indeed focus on the dorsal patches which are the most significant sessile hemocyte compartments in the larvae, containing some 1500 hemocytes per compartment.

(7) When they overexpress abrupt they show that hemocytes get relocalised to the dorsal vessel (Fig. 3). Interesting, but do they know why?

We see two phenotypes following Ab over-expression according to the level of Ab over-expression. With over-expression driven by hemocyte-specific GAL4 crossed to the P{EPgy2} misexpression insertion line P{EPgy2}ab^{EY09709} we see dispersal of hemocytes with no relocalisation to the dorsal vessel (DV). When crossed to *UAS-ab* transgenic line, higher levels of Ab over-expression can be obtained, and we observe both dispersal and relocalisation of hemocytes to the DV. It is possible that early expression of Ab, and at much higher levels than the normal physiological level that occurs in clear gut larvae is able to activate expression of cell adhesion molecules that enable hemocytes to aggregate at the DV. Our analysis of adhesion molecules shows that while Ab predominantly substantially repress adhesion molecules like NijA, it can slightly increase expression of factors like robo and scab (new Fig. 3A) that could be responsible for this relocalisation. However, we increasingly believe that relocalisation is an artefact of high-level over-expression and is not relevant to normal function of Ab in larval hemocytes. To reflect this, we have changed the emphasis of the section analysing Ab role in hemocytes, relying more on the loss-of-function experiments (*what Ab actually does do)* than over-expression experiments (*what Ab potentially can do*).

(8) The authors do not quantify the sessile patches in this experiment in a similar way to other experiments (e.g. in Fig. 5 they actually count sessile patches). Additionally, they do not examine dorsal vessel targeting in any other experiments. This is to say that their choice of analysis seems inconsistent.

We agree that the presentation of sessile compartment data in the first submission of the manuscript was not consistent throughout the manuscript which made comparisons between different experiments difficult or confusing. We have now rationalised how data is present utilising predominantly bubble graphs that quantify sessile patch number in at least 50 larvae from each experimental genotype/stage alongside individual exemplar images of single larvae. This format is used both for previously submitted experiments (Fig. 4A-N) as well as for additional experiments on MMPs (Fig. 4Q) and additional TF regulators (Fig. 6E). We have also added p values for T-test comparisons between control and experimental samples to show statistically significant variations. We hope this more consistent analysis will help to consolidate and simplify the message of the paper.

(9) The authors mention a few times that MMPs regulate ninjurins. Do they know if MMPs are also involved in controlling these sessile patches?

Again, this was a great comment! Thank you for the excellent suggestion! We have performed exactly these experiments to address MMP function and have included a new set of experiments (new Figure 4O-Q) showing that:

- i) The two fly MMPs, Mmp1 and Mmp2 are expressed in hemocytes, but the sole Timp is not (Fig. 4O)
- ii) An Mmp1 cleavage site occurs in the ECD of NijA (Fig. 4P)
- iii) Most importantly, that over-expression of Mmp1 (which can cleave NijA) results in loss of dorsal sessile hemocyte compartments, over-expression of Mmp2 (which does not cleave NijA) has no effect and over-expression of Timp stabilises dorsal sessile hemocyte compartments (Fig. 4Q).

© 2024. Published by The Company of Biologists under the terms of the Creative Commons Attribution License (https://creativecommons.org/licenses/by/4.0/).

Taken together these results show that that MMPs that can cleave Ninjurins disrupt dorsal sessile hemocyte compartments while inhibition of MMPs stabilises them.

Minor comment-
(10) Please add labels showing Figure numbers.
These have been added

(11) It is not good practice to use red/green overlays (https://www.ascb.org/science-news/how-to-makescientific-figures-accessible-to-readers-with-color-blindness/).
Thank you for the suggestion. We have changed all images from red/green overlays to magenta and green including single channels.

Reviewer 2:
(1) Authors suggest understanding the mechanism behind homing to sessile clusters will enable dissection of their role. This is easy to do now they have uncovered the mechanism - can they show some of the consequences of its manipulation?
This is a great question. We have tested whether preventing the dispersal of sessile clusters can influence immune responses in larvae. In the first submitted manuscript we had observed that over-expression of NijA in hemocytes stabilises sessile hemocyte clusters prevents their remodelling. We therefore investigated whether larvae in which NijA is over-expressed in hemocytes show reduced responses to model immune challenges. Three were assayed: i) sterile wounding of third instar larvae as well as ii) wounding with Gram-negative bacteria (*P. entomophila*), and iii) wounding with Gram-positive bacteria, (*E. faecalis*).

This has been included in a new Fig. 7A-C. We observed statistically significant reductions in survival of larvae over-expressing NijA in hemocytes in response to all these challenges, ranging from a small, but significant, reduction in survival in response to sterile injury (new Fig. 7A), to much greater reductions in survival following septic injury with either P. entomophila (new Fig. 7B) or E. faecalis (new Fig. 7C).

(2) What are the point of these clusters - is proliferation affected if this mechanism is perturbed?
In our original submitted draft we included proliferation data for control larvae at different stages, comparing proliferation in circulating hemocytes with proliferation in sessile compartments and also following Ab expression (Ab disrupts the dorsal sessile compartments). The analysis used histone H3S10phos staining to detect cells in M-phase and determined the percentage dividing cells in each population. Our initial assumption was that the dorsal sessile clusters could be a niche that drives proliferation, but we observed that proliferation rates where consistently lower in sessile compartments than in circulation. Moreover, over-expression of Ab did not suppress proliferation as mitotic index was fairly similar to that observed in foraging stage circulating hemocytes. The most significant impact was the small rise in ecdysone that occurs in wandering stage larvae that reduced proliferation rates both in sessile and circulating hemocytes. Our conclusion was that targeting to sessile hemocyte clusters does not facilitate proliferation, if anything proliferation rates in clusters is slightly lower than in circulation.

For our revised manuscript we thought long about including this data and eventually decided to leave it out. Reviewers felt that the message of the original submission was not focused enough, and we felt that continued inclusion of this paper diluted the main message of the paper. I am happy to include this data should you feel it contributes to the message of the paper and have however included it below for information

© 2024. Published by The Company of Biologists under the terms of the Creative Commons Attribution License (https://creativecommons.org/licenses/by/4.0/).

Table S1: Hemocyte proliferation index at various stages of larval development

Genotype assayed	*Larval stage/hemocyte population*	*H3S10p$^-$ /GFP$^+$ (%)*	*H3S10p$^-$ /GFP$^+$ (%)*	*Hemocytes scored*
Pxn-GAL4, UAS-GFP x w^{1118}	Feeding 3rd instar/circulating	94.1	5.9	322
	Feeding 3rd instar/sessile	97.8	2.2	698
	Wandering 3rd instar/circulating	99.3	0.7	433
	Wandering 3rd instar/sessile	99.8	0.2	466
Pxn-GAL4, UAS-GFP x UAS-ab	Wandering 3rd instar/circulating	95.9	4.1	317
	Wandering 3rd instar/dorsal vessel	96.0	4.0	347

(3) In the discussion authors suggest other situations in which clusters may disperse - can they look at one or more of these to address how critical/relevant this mechanism is? (immune challenge, environmental stress, tumours). Addressing the importance and relevance of this mechanism would go a long way to strengthening the paper and broaden the range of interest in this work.

Again, good question. We have looked at an additional instance in which we could trigger hemocyte dispersal. This has been already described in responses to reviewer 1 comments where we examine the effect of MMPs on clusters. Expression of expression of Mmp1 is elevated after Drosophila immune challenge. We have shown with a new set of experiments (new Figure 4O-Q) that:

i) The two fly MMPs, Mmp1 and Mmp2 are expressed in hemocytes, but the sole Timp is not (Fig. 4O)
ii) An Mmp1 cleavage site occurs in the ECD of NijA (Fig. 4P)
iii) Most importantly, that over-expression of Mmp1 (which can cleave NijA) results in loss of dorsal sessile hemocyte compartments, over-expression of Mmp2 (which does not cleave NijA) has no effect and over-expression of Timp stabilises dorsal sessile hemocyte compartments (Fig. 4Q).

Taken together these results show that that MMPs that can cleave Ninjurins disrupt dorsal sessile hemocyte compartments while inhibition of MMPs stabilises them

(4) Does this mechanism influence hemocyte biology at other ecdysone peaks? It looks there is low level of Abrupt expression in hemocytes - could this relate to previous peaks via protein perdurance?

Lynne Riddiford spent many years carefully quantitating ecdysteroid levels across insect development from egg, through larvae to pupae both in *Manduca* and *Drosophila*. Recent LC-MS/MS analysis on Drosophila by Lavrynenko and colleagues (2015) essentially confirm these trends. It is true that there are other ecdysone peaks during development prior to the late larval/prepupal peak that triggers hemocyte dispersal but these are dwarfed in magnitude by the late larval/prepupal peak. There is a small peak at the end embryo stage when larvae hatches, followed by small peaks at each larval moult: L1-L2, and L2-L3. These are followed by a small peak during 3rd instar that triggers the wandering stage (128hrs in the figure from Lavrynenko and colleagues (2015) below) and then the large peak at late larval/prepupal stages (144hrs below) that we have used to select clear-gut stage larvae.

NOTE: *Figure provided for reviewer has been removed. It showed Figure 2A from Lavrynenko et al (2015) The ecdysteroidome of Drosophila: influence of diet and development. Development. 142, 3758-3768. (DOI: 10.1242/dev.124982).*

In embryo stages, hemocytes migrate within tissue and do not occur in circulation so there is not an analogous sessile hemocyte compartment system. However, we do detect Ab expression in late stage embryonic hemocytes. For larval stages, previous analyses by our group (Stofanko et al. 2008) suggests that dorsal sessile hemocyte clusters are not obvious during 1st instar larval stages

© 2024. Published by The Company of Biologists under the terms of the Creative Commons Attribution License (https://creativecommons.org/licenses/by/4.0/).

(probably due to low hemocyte number) and only become manifest during second larval instar as hemocyte numbers increase. We have not examined whether there is a similar disruption of compartments at the L2-L3 moult or looked at Ab expression. However, we have examined patches and Ab expression in wandering larvae, after the small mid L3 peak. At these stages we do not see any disruption of sessile compartments or Ab expression unlike the response that occurs at the late larval -prepupal peak. We speculate that the response of Ab may be sensitive to levels of ecdysone and that necessary concentrations to trigger activation are only attained during the late larval peak.

(5) Does interfering with Ecdysone signalling block hemocyte relocalisations in vivo? If already known these papers should be cited, if not, this should be done. Given the role of Ab in other cell types can authors show that Ab expression is affected by EcR signalling in a hemocyte-autonomous manner (e.g. using Dominant negative or active versions of EcR expressed specifically in hemocytes)?

We have tried to use DN versions of EcR to address this. Interpretation of experiments in which these DN-EcR versions are driven in hemocytes throughout larval stages is somewhat complicated by the fact that constitutive expression of DN-EcR versions affect not only EcR activation by ecdysteroids at the late larval ecdysone pulse but also earlier repression in the absence of ligand. Attempts to refine the period during which these are expressed by using Gal80ts-mediated expression control by upshift to 29C is hampered by the fact that dorsal sessile compartments are sensitive to growth at higher temperatures.

(6) On page 6 authors may wish to cite references that back up correlation of ecdysone signalling levels with the presence of dye in the larval gut/changes in larval behaviour (I realise these are in the methods).

We have included additional references to ecdysone levels including more recent LC-MS/MS analyses (Lavrynenko et al. (2015)) see Reviewer 2: point 4 above. We have also followed the suggestion of Reviewer 1:point 5 and performed timecourse analysis of our staged collections to define time to pupariation for these collections and determine how these collections and the larval gut/changes in larval behaviour upon which they are based correlate with ecdysone peaks. These additions and text changes are described in detail in Reviewer 2: point 4 above.

(7) Why is the activation of the LacZ reporter so heterogeneous in the larval hemocyte population? Can authors show in vivo staining to demonstrate that it is the LacZ positive cells (or a live reporter) specifically leave clusters.

We thank the reviewer for highlighting a problem with the slide preparation and imaging methods we used to generate the figures in the initial submission. These were derived from confocal scans in a single Z-plane. However, the hemocyte preparation methods used to generate these slides (hemocytes are allowed to settle under gravity and attach to the glass slide surface) hemocytes do not occupy a single uniform Z-plane but rather can flatten onto the slide surface or alternatively remain loosely attached to the slide. If a single confocal Z section is used it results in some hemocytes being out of plane with apparently lower expression than hemocytes that are in the plane of imaging. We have remedied this by repeating these experiments, generating confocal Z-stacks and then generating projections that more accurately show expression in all cells. This data is included in a new Figure 1D-I with image quantitation performed in Supplementary Figure S2.

(8) Where else are the Ninjurin's expressed? The model in Figure 7 suggests hemocytes are using Ninjurin's to attach to tissue as well as each other. This mechanism may account for hc-hc adhesion but do Ninjurin's expressed elsewhere help this process? If it is just hc-hc adhesion this does not explain localisation in these specific clusters.

This is a great question. The reviewer quite correctly points out the hemocyte Ninjurin expression enables hemocytes to adhere to each other but alone does not explain why hemocytes go to specific clusters. To determine whether there non-hemocyte tissue that underlies the clusters express Ninjurins we performed live imaging of larvae that contain the hemocyte *HmlΔ-DsRed.NLS* reporter as well as *NijA-GFP* gene-trap. Prior to this work to define potential cues that could be used to organise the dorsal sessile hemocyte clusters we also performed live imaging of larvae that contain the hemocyte *HmlΔ-DsRed.NLS* reporter as well as muscle reporters (Zasp52-GFP; New Figure 1M-P) and pericardial cell reporters (New Fig. 1Q-T). Examination of *HmlΔ-DsRed.NLS*; *NijA-GFP* larvae showed that for both dorsal sessile hemocyte clusters (New Figure 3K-N) and lateral hemocyte clusters (New Figure 3O-R) NijA-GFP could be detected in the hemocytes in the clusters

© 2024. Published by The Company of Biologists under the terms of the Creative Commons Attribution License (https://creativecommons.org/licenses/by/4.0/).

but also in non-hemocyte tissue associated with the cluster. We would argue that, as the reviewer suggests, Ninjurins are expressed in hemocytes but also in non-hemocyte tissue to which they are associated. This together gives rise to the clusters that are observed. Accordingly, we have added the following text to the results: "*To provide evidence for this, we performed live imaging of HmlΔ-DsRed.NLS larvae that also contain the NijA-GFP gene-trap. This revealed that NijA expression could be detected in hemocytes that comprise the dorsal sessile compartment (3K-N, open arrowheads) but was also observed in tissue that underlies these hemocytes (3N, closed arrowheads). NijA expression was not detected in the flanking DO1 and DA1 muscles. Similarly lateral sessile hemocyte clusters were also observed both to express NijA and be associated with NijA-expressing non-hemocyte tissue (Fig. 3O-R). Taken together these results suggest that localised Ninjurin expression in non-hemocyte tissue may facilitate hemocyte targeting to establish SHCs.*"

(9) High resolution (in z)/live imaging of some of these clusters as they disperse may help develop the model further, though I recognise those would be technically challenging experiments.
This is our dream experiment. For many years, since we first observed these structures, we have attempted to establish live imaging systems that would allow us to perform live imaging of these structures over extended time periods to either examine the time course of recruitment of hemocytes into these structures or as suggested by the reviewer, the time course of their dispersal. In fact, some of the imaging data in the paper – Supplementary Figure 3 (hemocyte numbers in compartments) and the organisation of the compartments (New Figures 1M-T; and 3K-R) - emerged out of two different attempts to do exactly this, either using Zoom confocal microscopy or standard confocal microscopy on anesthetised and immobilised larvae.

However, the main challenge is that neither of these approaches allow long-term imaging of the clusters in larvae. A point we tried to make in the first submission, was that the bulk of the Ninjurin extracellular domain is composed of intrinsically-disordered domains (IDRs). IDRs can facilitate clustering to produce liquid-liquid phase-separated condensates. We argued that this is important for enabling clustering of cells but that phase-separated condensates also exhibit the properties of hysteresis – small changes in environmental conditions cause dramatic changes in their ability to form or to be destabilised. On the suggestion of Reviewer 1 (Reviewer 1: point 3 above) we have removed this discussion of Ninjurins, IDRs or phases as it detracts from the main point of the paper. But I think it is worth repeating here in the context of live imaging. These hysteretic properties of the phase means that Ninjurin-directed interactions are sensitive to small changes in environment. We think this is useful in enabling release of hemocytes to deal with threats. But, it does mean that the clusters are acutely sensitive to manipulations and changes in environment that make long term, stable imaging of the type required for this experiment possible.

(10) Relocalisation is not quantified in the early figures, though comparable genotypes are quantified in Figure 5.
As mentioned in point 7 above we believe that the relocalisation phenotype observed is an artefact of abnormally high levels of Ab expression. While an interesting phenotype we would argue that the Ab hemocyte loss of function phenotypes are more important in illuminating roles of Ab in hemocytes and have removed the original Figure 3G-J from the manuscript.

(11) Where have the hemocytes gone in Fig 3D - can we be sure the hemocytes have not largely been killed off due to this misexpression strategy?
No, the hemocytes are not killed off. In point 2 (Table S1) above we show that we were able to recover hemocytes both from circulation and from the new accumulations along the dorsal vessel. They exhibited proliferation rates and were recovered in numbers similar to control larvae. As we argue in point 7 above, very high levels of Ab over-expression causes two effects. Firstly, dorsal and lateral sessile hemocyte clusters are dispersed. Secondly some of the hemocytes start to accumulate along the dorsal vessel.
(12) Whole larvae should be shown alongside quantification of total hemocyte numbers here.
We have analysed hemocyte numbers and see no reduction in number or proliferation rates point 2 (Table S1). If anything, proliferation rates are increased.

(13) Why does Figure panel 3D not look more like 3C if the cells are dispersed? Why do we see this weird localisation at the dorsal vessel rather dispersing as per 3C? Total numbers of cells is important as fewer hemocytes will mean fewer available to stick in these clusters which will in

© 2024. Published by The Company of Biologists under the terms of the Creative Commons Attribution License (https://creativecommons.org/licenses/by/4.0/).

turn make them less obvious (and may also provide insight into importance with respect to haematopoiesis).
As argued point 7 above we believe that the relocalisation phenotype observed is an artefact of abnormally high levels of Ab expression. While an interesting phenotype, or as described by the reviewer "weird localisation" we would argue that the Ab hemocyte loss of function phenotypes are more important in illuminating roles of Ab in hemocytes and have removed the original Figure 3G-J from the manuscript.

(14) Scoring of clusters is not explained in detail and may be subjective. How are dorsal patches defined (how big do they need to be to be scored?) I can't see how this was done in the methods. Was the scoring of what appears to be a slightly subjective phenotype done on blinded samples?
We apologise for this oversight. These details are now provided in an expanded section of the materials and methods section. It is worth noting that there is a trade-off between speed and resolution and dorsal SHC stability when imaging. For scoring dorsal SHCs we have used stereo fluorescence microscopes as it is less harsh and can be performed rapidly, helping to preserve dorsal SHCs in all larvae of the collection.

As we discussed in response to comment 9 above, our experience is that preparation for higher resolution imaging methods like confocal or zoom microscopy can lead to the loss of dorsal SHCs as these structures seem to be intrinsically programmed to be sensitive to changes in environment. While we obviously can maintain structures in enough larvae to get high quality exemplar data (as shown in Fig1 and Fig 3, it is difficult to maintain these structures in all larvae or for long periods of time. As such high-resolution imaging methods are not appropriate for performing quantitative analysis on dorsal SHCs where all larvae are to be analysed.

Instead, we have used stereo fluorescence microscopes to analyse larvae that have not been anesthetised or immobilised, with short times between collection to scoring which allows better preservation of dorsal SHC integrity. However, the resolution of the stereo microscope and the fact that larvae are not immobilised makes it difficult to set absolute thresholds on hemocyte number that need to be present for accumulations to qualify as dorsal SHCs. We are simply scoring for accumulations of GFP-positive hemocytes at the dorsal midline of each larval segment. It is important to stress, however, that if we do take larvae that have been sorted and viewed and scored using a stereomicroscope, and then prepare them for Zoom confocal microscopy and determine hemocyte numbers in dorsal SHCs we can infer that dorsal SHCs contain maximally 1500 hemocytes and at least 300 hemocytes per cluster.

To provide more detail on the methods used for sorting and then scoring larvae for dorsal SHCs (including details on blinding) we have expanded on the relevant part of the Materials and Methods section. Specifically, a section "*Staging of 3rd instar larvae and scoring of dorsal sessile compartments*" has been added that includes the following text:

"For staging, larvae were raised on food supplemented with 0.05% bromophenol blue (Maroni and Stamey, 1983). By selecting larvae on the basis of their foraging behaviour and colour of the gut, third instar larvae could be staged as i) "foraging" stage larvae that have blue guts and are continually feeding, ii) "wandering" stage larvae that have blue guts and moving in an out of the media, and iii) "clear gut" stage larvae which have evacuated the larval gut and have left the medium. Analysing time to pupariation of these collection windows (Fig. S1) puts these stages at i) >30 hours before pupariation, ii) 12-24 hr before pupariation and iii) 4-6 hr before pupariation. Staging was performed by one investigator with collections transferred to numbered 35mm Petri dishes for each genotype and stage and then scored blind for dorsal SHCs by a second investigator. Each experiment typically comprised multiple genotypes and stages alongside GAL4-driver crosses with control w^{1118} flies, assayed side by side. The second investigator visualised and scored dorsal SHC number in each larva by manual inspection using an Olympus SZX12 stereo fluorescence microscope, scoring each larva as having between 0-6 dorsal SHCs. The hemocyte GAL4 driver lines contain UAS-GFP transgenes that enabling dorsal SHCs to be detected as GFP foci along the dorsal midline of larvae. Data of numbers of larvae from each collection and respective dorsal SHC class 0-6 were then collated and decoded by the first investigator to apply data to the relevant genotype and stage."

© 2024. Published by The Company of Biologists under the terms of the Creative Commons Attribution License (https://creativecommons.org/licenses/by/4.0/).

(15) The report identifies a number of transcription factors and motifs potentially involved but their importance is not addressed experimentally. Can the role of one or more of these be investigated with respect to relocalisation of hemocytes and so test/support the bioinformatic/molecular profiling data? To address the importance of the motifs likely requires enhancer analysis/new transgenics and is probably beyond the scope of this report (unless the authors get very lucky with publicly available mutants) so the role of the transcription factors seems easiest to address in their imaging of clusters assay. I think this would add to the story and certainly round it off nicely.

We agree with the reviewer on this point. This point was also raised by Reviewer 1 (reviewer 1: point 1) above. To address this, we have performed exactly the type of functional studies requested by the reviewer. It has not been possible to perform enhancer-bashing experiments. But we have explored roles of these factors in cluster formation using inducible RNAi-mediated knockdown and over-expression and characterized expression relative to the larval stages. To reiterate from reviewer 1:point 1 above:

We have strengthened this section by performing additional experiments that include:

- *i) Comparison of expression of these transcription factors to known hemocyte regulators to confirm that their expression is at the level of other factors with demonstrated function (new Fig. 6B)*
- *ii) Analysis of expression profile of these TFs across the developmental time course from feeding, wandering to clear gut larval stages confirming that expression of these factors mirrors expression of Ab and expression increases occur when hemocytes are dispersed from sessile compartments (new Fig. 6C)*
- *iii) Use of GFP fusions with these TFs to confirm that they are translated and nuclear-localised in hemocytes when sessile compartments disperse (new Fig. 6D)*
- *iv) Performed hemocyte-specific over-pression and also RNAi to demonstrate that all TFs are required for hemocyte dispersal from sessile compartments (new Fig. 6E), confirming function. Over-expression demonstrates that unlike Ab which is sufficient to cause early hemocyte dispersal none of these TFs can trigger complete dispersal when over-expressed alone (new Fig. 6E).*

We trust these additional work helps to validate this network of factors as not only being expressed but also being functionally required for this process.

(16) Authors claim no expression of LacZ in Fig1 D,G but green channel fluorescence clearly visible. This requires some sort of negative control to make this statement as this data could also be interpreted as low/high levels of LacZ. Also this is not uniform across all hemocytes so some quantification of proportions would be helpful.

We thank the reviewer for pointing this out. In response also to comments from reviewer 1 (reviewer 1:point 7) we repeated these experiments, generating confocal Z-stacks and then generating projections that more accurately show expression in all cells. This data is included in a new Figure 1D-I. We have also performed image quantitation of multiple images per genotype and added these as Supplementary Figure S2. This includes comparison between signals in foraging and wandering larvae (S.Fig. S2A) and between wandering and clear-gut stage larvae (S. Fig. S2B). This confirms statistically significant differences in lacZ signal between foraging and wandering stage hemocytes and wandering and clear gut stage larvae and also enables the distribution in signal to be compared. The important take-home message of this analysis is the significant upregulation of reporter expression that occurs between wandering and clear gut stages, that corresponds with the dispersal of dorsal SHCs, and that there is no overlap in expression between any of the hemocytes from wandering and clear gut stages indicating that the increase in reporter expression occurs in all hemocytes of clear-gut stage larvae.

(17) Fig. 2 – again some Abrupt staining appears present in absence of high levels of LacZ. Correlations between Abrupt and LacZ should be quantified in some way for reproducibility.

Quantitation of signals has been performed for both LacZ (see point 16 above and S.Fig 2A). Quantitation of Ab signal has also been performed and added as new data in Figure 2 (Fig 2G) that demonstrates statistically significant increase in nuclear Ab in clear gut stage hemocytes when there is also statistically significant increase in EcRE-lacZ reporter expression (point 16 above).

© 2024. Published by The Company of Biologists under the terms of the Creative Commons Attribution License (https://creativecommons.org/licenses/by/4.0/).

(18) Given Fig 2L's result authors may want to consider perdurance of protein from previous bouts of ecdysone signalling. At the end of page 7 it is stated that Ab is absent; this should be rephrased as low levels in comparison to when ecdysone signalling is intact.
Fig. 2L showed semi-quantitative RT-PCR analysis of Ab transcript at feeding/foraging and clear gut stages and indicated that Ab transcript was **absent** at these stages while up-regulated at clear gut stages. However, there is a weakness with this analysis that it was performed by "end-point" RT-PCR where a single PCR cycle number is used, and which can magnify apparent differences in transcript levels. To address this, we have performed this experiment again using real-time RT-PCR analysis which shows that *ab* transcript levels at clear-gut stages are 140-fold higher than at foraging/feeding stage. This data has been added as a new Fig. 2H. We have also changed the relevant text in the results section to read: *"Anti-Ab staining failed to detect nuclear Ab in foraging stage hemocytes (Fig, 2A,B) but detected Ab in clear gut stage hemocytes when hemocytes disperse (Fig, 2C,D), consistent with Ab regulating hemocyte dispersal."* I think I understand that reviewer's point, how can one be sure that protein/mRNA truly is absent as one is simply not detecting it using the thresholds applied.

(19) I cannot find Figure S1 is cited in the text. Why are different regions of the embryo used to show absence of/expression of Abrupt in Fig S1 E and F? The same region of the embryo should be used.
We have made a number of changes to the supplemental material to help address concerns raised by both reviewers regarding the focus of the manuscript. The original Figure S1 has now been removed from the revised manuscript as it is not directly relevant to the main message of the paper.

(20) Figure S2 - I think there is GFP expression when Gal80[ts] flies are raised at 18oC. Though it is definitely reduced (the presence of GFP would be more obvious if a more restricted band pass filter had been used to image larvae). Authors should probably modify statements as it looks like (according to GFP's presence) that low levels of Ab may be present despite the Gal80[ts] strategy at 18oC.
We have shifted the focus of the manuscript from Ab over-expression to Ab loss-of-function phenotypes that we feel more accurately capture biological requirements for Ab in SHCs. Accordingly, these Gal80ts timecourse experiments have been removed.

(21) Some of the references are not formatted correctly.
References are now checked for formatting.

(22) Fig 2K cited ahead of G-J - move panel over to the left so K becomes 2G to help reader?6
We have extensively reorganised Figure 2 which has addressed this issue

(23) P8 2nd sentence - missing "we" from start of sentence
Extensive changes to the text have addressed this comment

(24) Figure 1A and Figure 3A look to be the same image. Can authors use different images?
We have extensively reorganised Figure 3 which has addressed this issue

(25) The 3D structures are beautiful and interesting but it's not clear how this information progresses the main story here.
Agreed, these have been removed to focus on the core message of the manuscript.

(26) Perhaps this could re-written a little to this data in to the overarching thread more clearly as, currently, it feels like a slightly superfluous (but pretty) addition.
Structures have been removed to focus on the core message of the manuscript.

(27) The analysis of bled hemocytes looks robust, but can authors confirm how well their methods release cells attached to the body wall as this may influence magnitude of the effects they see?
Recovery of circulating hemocytes by our preparation methods appears to be robust. The driver behind the development and optimisation of our hemocyte preparation methods was to generate sufficient hemocytes to be able to perform ChIP-seq chromatin profiling experiments. The sensitivity of initial ChIP-seq methods required $5x10^6$ cells. We have been able to generate at least

© 2024. Published by The Company of Biologists under the terms of the Creative Commons Attribution License (https://creativecommons.org/licenses/by/4.0/).

these cell numbers by "ripping" 1000 larvae suggesting that we can obtain on average at least 5000 hemocytes per larvae. For immunofluorescence experiments reported here we typically use far fewer larvae per prep, between 5-15 larvae. Obviously developmental stage has an impact as at foraging and wandering stages a proportion of hemocytes are in SHCs and do not detach from the cuticle while as clear gut stages SHCs no longer occur and all hemocytes are in circulation and can be recovered.

(28) Image in 5B is not as convincing as in other figure panels for the equivalent control.
The figures and quantitative an analysis for over-expression and loss-of-function phenotypes have been revamped as a new Figure 4 that we hope addressed a number of reviewer comments regarding consistency in data presented and analysis methods. The new figure corresponding to Fig 5B is Figure 4B that should address this concern.

(29) Page 11 "snap-back darns" – "RNAs"?
Corrected.

(30) Panel 6E needs more explanation in the text/legend as for those not familiar with iRegulon this is very hard to interpret.
We thank the reviewer for this comment. Familiarity with specialised jargon and techniques of a field can lead one to make the mistake of assuming all readers will have detailed knowledge of what can be niche methods. We have attempted to address this concern, while at the same time maintaining brevity of the manuscript, by adding an expanded section to the corresponding figure legend for this image, which is now Figure 5E. Specifically we have added the following text to the figure legend for Figure 5:

"*iRegulon analysis of the* 5kb upstream cis-regulatory regions *of Ab ChIP target genes identified statistically significant enrichment of known DNA-binding sites for the fly TFs Gem, Ewg, Blimp-1 and Hr3 in the regulatory regions of Ab target genes. For each identified TF, network analysis was performed to show the extent of overlap (and co-regulation) in Ab targets between each TF. Individual TFs are displayed as rectangular nodes coloured according to TF identity (Gem:pink, Ewg:green, Blimp-1:orange and Hr3:purple). Individual Ab target genes are displayed as oval nodes (coloured blue) and connections between TFs and targets are displayed as edges (lines) coloured according to the TF. Targets that are regulated by more than one TF lie on the circle, those that are regulated by a single TF lie outside the circle and are labelled according to the single TF that binds the regulatory region.*"

(31) Figure S3 – no legend. Can some non-expressed TFs be shown as a comparison?
We have reduced the amount of Supplemental data provided for the manuscript and have removed Figure S3. The data that was in Figure S3 is now contained entirely in manuscript Figure 6A. To provide a more comprehensive comparison of how mRNA-seq and CAGE-seq expression of our identified TFs compares with other TFs, both expressed and non-expressed we have included the plot in Figure 6B. This compares normalized CAGE-seq read rpm (reads per million) and mRNA-seq tpm (transcripts per million) for all known fly TFs in 3rd instar larvae. TFs identified in this study, as well as TFs known from previous investigations to be regulate hemocyte (at some stage in development) are labelled. This plot gives a visual comparison of how expression of identified TFs compares with known factors and also expression relative to other TFs for which no previous data exist, but which show low expression in our study. Interestingly there is some overlap between the two sets as for example gcm (which has been shown to counteract Tl-induced inflammation is one of the fly TFs with lowest expression in hemocytes.

(32) Check all supplementary tables and figures are cited in the text. I think some are missing.
We have made extensive changes to the supplemental material, adding timecourse analysis of staged larval collections to confirm timing relative to ecdysone peaks. However, we have removed material that is not directly related to the core message of the paper. This should help address this comment but also improve the focus of the paper, a concern for both reviewers.

Resubmission

© 2024. Published by The Company of Biologists under the terms of the Creative Commons Attribution License (https://creativecommons.org/licenses/by/4.0/).

First decision letter

MS ID#: DEVELOP/2024/202977

MS TITLE: Abrupt-mediated control of Ninjurins regulates Drosophila sessile hemocyte compartments

AUTHORS: So Yeon Kwon, Kimberley Chan, Martin Stofanko, Ka Hei Chan, and Paul Badenhorst

I apologise for the delay in getting back to you do you difficulties finding available reviewers. I have now received all the referees reports on the above manuscript, and have reached a decision. The referees' comments are appended below, or you can access them online: please go to BenchPressand click on the 'Manuscripts with Decisions' queue in the Author Area.

The overall evaluation is positive and we would like to publish a revised manuscript in Development, provided that the referees' comments can be satisfactorily addressed. The reviewers make useful suggestions to improve the manuscript. It is my opinion that the revision should not require any further experiment, so you may focus on comments of reviewer 2 that do not require experiments. Please attend to the reviewers' comments in your revised manuscript and detail them in your point-by-point response. If you do not agree with any of their criticisms or suggestions explain clearly why this is so. If it would be helpful, you are welcome to contact us to discuss your revision in greater detail. Please send us a point-by-point response indicating your plans for addressing the referees’ comments, and we will look over this and provide further guidance.

Reviewer 1

Advance summary and potential significance to field

In my view this is a really high quality and very thorough MS focussing on the role of a fairly novel cell adhesion protein, ninjurin, in targeting, and holding a third of all Drosophila larval hemocytes to aggregations, called SHCs, on the inner surface of the cuticle. At pupariation these aggregations disperse and this can be blocked by stabilisation of ninjurin; inactivation, by contrast, causes premature dispersal.
In a previous study they had identified the transcription factor, Abrupt, as a regulator of third instar hemocyte patterning, and ninjurin is the output of an “Abrupt over expression” screen to identify potential targets of Abrupt that might be the downstream effectors.
In addition to the very clear genetic and experimental data to demonstrate the above, the authors also show very clear CHiP Seq data to confirm a direct interaction between Abrupt and upstream regulatory regions of ninjurin, and report experiments showing how several other transcription factors, gem, ewg, hr3 and blimp-1, work alongside Abrupt in this regulation. And finally, they show a physiological relevance for release of hemocytes from SHCs by blocking release through overexpression of ninjurin, and showing significantly worse survival after wounding and infection insults.

Comments for the author

To me this body of work appears thorough and complete as is. In places I got confused by the developmental staging of larvae (foraging, wandering) and the dispersal, migration of hemocytes; on page 7 there are several places where a niave reader may get lost in ambiguity. I was also a little unclear whether the authors showed any data comparing expression levels of Abrupt and ninjurin in the third of hemocytes in SHCs versus the two thirds of their siblings that feely circulate, prior to SHC dispersal (at the clear gut stage?) I may have misread but the switch on of Abrupt seemed temporal, rather than spatial? Are the circulating hemocytes at early stages not adhering because they don’t express ninjirin, or for some other reason?
As I finished reading the Results section of this MS, I was expecting to have to suggest that the authors add some text in the Discussion to offer insights beyond Drosophila, but no need. We are told that ninjirins indeed have several interesting functions in mammalian inflammation that will make these findings in the fly even more significant for others. Nice paper.

© 2024. Published by The Company of Biologists under the terms of the Creative Commons Attribution License (https://creativecommons.org/licenses/by/4.0/).

Reviewer 2

Advance summary and potential significance to field

In this study, Kwon et al. explore the molecular mechanisms underlying the developmentally regulated movements of Drosophila larval hemocytes to sessile patches. The authors show that the transcriptional repressor Abrupt is part of a transcription factor network involved in hemocyte dispersal before pupariation. Using several sequencing methods, they show that Abrupt binds to and represses the promoter of two Ninjurin genes. Abrupt upregulation and Ninjurin downregulation are necessary and sufficient to disperse hemocytes from sessile compartments.
In this comprehensive study, the authors describe a detailed timeline of hemocyte detachment between larval and pupal stages. They also provide compelling evidence that Ab orchestrates the collective detachment of hemocytes by downregulating the homophilic cell adhesion molecules Ninjurin. Their work provides a simple mechanism explaining how hemocyte disperse.
I have however four majors suggestion and some minor comments that are critical to reinforce the conclusion of this manuscript.

Comments for the author

Major suggestions
1- It has been reported that sessile hemocytes detach when vortexing or brushing the larvae. Similarly, hemocytes are expected to disperse upon systemic infection. It would be interesting to see how the hemocytes overexpressing or knocked-down for Ninj behave in these contexts.
2- The receptors Eater and Multiplexin have been implicated in the formation of sessile patches (Bretscher et al., 2015, Csordars et al., 2020). It is surprising that these important articles are not mentioned and discussed in the present article. Could Ab be involved in the regulation of Eater? How to reconcile the data from these papers and the present observation implying a role of ninjurin?
3- A study analyzing the role of Ninjurin A (Broderick et al., 2012) is not mentioned in apoptosis. It should be discussed.
4- The NijA null mutant should be used to address the role of NijA in hemocyte sessility

Minor suggestions
1) The recent study showing the migration of hemocytes, notably crystal cells, to sessile patches upon H2O2 should be mentioned (Shin et al., Nature 2024). Thus, sessile patches could have a role in hemocyte/hemolymph oxygenation.
2) Top of page 5: what are da neurons?
3) Bottom of page 7 ‘determining that each compartment’ Explicit what are those compartments.
4) Page 13: Stofanko is not the only ref revealing that hemocytes leave sessile patches upon challenge.
5) Page 15: Add Stephenson et al., 2022 to indicate that hemocyte are critical during metamorphosis
6) There is no method section describing the statistics used in this study. It is unclear how the t-test was applied on different categories (number of patches) at the same time. A different test might be more suited. There is no statistical analysis on Fig2H. The Y axis legend is missing in Fig2M.
7) Survival to bacterial infection can vary drastically across genetic backgrounds. Therefore, it is necessary to also test the UAS line crossed to + in the infection experiments.
8) It is stated that gem overexpression has no impact on hemocyte homing but Figure 6E shows a significant difference, could you explain this discrepancy?
9) Purified LPS does not activate the Drosophila immune response, therefore it is misleading to discuss together response to LPS and bacterial infection (see Leulier et al., 2003, Kaneko et al., 2004). This section might be rephrased to avoid confusion.

© 2024. Published by The Company of Biologists under the terms of the Creative Commons Attribution License (https://creativecommons.org/licenses/by/4.0/).

First revision

Author response to reviewers' comments

Response to reviewers

We would like to express our gratitude to the reviewers for both their positive comments on our manuscript. Below, please find a point-by-point reply to the reviewer's comments (reviewer comments in italics). We have taken the liberty of numbering each of the reviewers comments to allow easier cross-referencing between responses.

Reviewer 1 Comments for the Author:

(1) To me this body of work appears thorough and complete as is.

We thank this reviewer for their positive assessment of our work.

(2) In places I got confused by the developmental staging of larvae (foraging, wandering) and the dispersal, migration of hemocytes; on page 7 there are several places where a niave reader may get lost in ambiguity.

We thank the reviewer for pointing this out. We have attempted make this clearer to non-specialist reader by making changes to the text that we hope clarify the differences between the stages and makes the relevance of these explicit. Additions include:

i. Adding an additional schematic to S. Fig 1 (generating a new S. Fig. 1A) that summarizes the behavioral and gut characteristics of the foraging, wandering and clear gut larvae together with summary schematics of the organisation of dorsal sessile compartments at these various stages with the times relative to pupariation indicated. We debated adding this schematic to Fig. 1 (making a new Fig. 1A) so that this information was explicit and available from the very start of the results but felt that this overloaded what was an already busy figure. Happy to take suggestions on this.

ii. Text changes to paragraph 1-2 of the results section that place the three 3rd instar stages (foraging, wandering and clear gut) in the context of the broader fly developmental programme and metamorphosis.

iii. Addition of text to S. Fig. 1 legend that describe the three distinct changes in locomotor and feeding behaviour that mark and distinguish the last 30hrs of 3rd larval instar stage.

(3) I was also a little unclear whether the authors showed any data comparing expression levels of Abrupt and ninjurin in the third of hemocytes in SHCs versus the two thirds of their siblings that feely circulate, prior to SHC dispersal (at the clear gut stage?)

Obtaining a hemocyte preparation that is exclusively made of only of circulating hemocytes is easier said than done. We suspect that an integral feature of SHCs is that they are programmed to be dispersed in response to challenge and/or perturbation.

The extracellular domain of Ninjurins (especially obvious for NijA) is largely composed of intrinsically disordered domains (IDRs). An emerging body of research shows that IDRs can facilitate protein clustering and the formation of liquid-liquid phase-separated condensates. In previous drafts of this manuscript, we had argued that that such phases may enable SHC formation. However, phase-separated condensates display exquisite sensitivity to changes in physicochemical conditions, with small changes in conditions (eg temperature, pH etc) leading to large shifts in the propensity to form or disrupt condensates. This work is still at a very early stage, but if the adhesive function of Ninjurins works through the formation of phase-separated condensates, such hysteretic properties for IDRs and resultant phases could enable programmed instability in adhesive complexes and explain the sensitivity of SHCs to perturbation.

Thus, while methods have been developed to ensure that all hemocytes can be dislodged from SHCs to enable the recovery of all hemocytes from larvae (for example centrifuging larvae or brushing cuticle with paintbrushes, needles etc), it is actually quite difficult to make a hemocyte preparation from larvae that one can categorically state is exclusively composed of circulating hemocytes with no contamination from sessile (SHCs). Thus, most of our immunostaining and mRNA expression analysis of hemocytes from 3rd instar larvae

© 2024. Published by The Company of Biologists under the terms of the Creative Commons Attribution License (https://creativecommons.org/licenses/by/4.0/).

reflects data from hemocytes that are predominantly circulating but with sessile contribution.

In contrast, we have developed methods that enable sections of cuticle with attached SHCs to be isolated and the SHCs then dislodged enabling purification of sessile hemocytes. However, these preparation methods are laborious to say the least and yields are insufficient for routine mRNA quantitation. We however used these to perform anti-Ab immunostaining, that indicates no differences in Ab expression (absent) in hemocytes isolated directly from the SHCs or from whole animals at the wandering stages. Obviously in clear gut stages the hemocytes are dislodged so distinction between those that had once been in compartments and those that had been in circulation is lost, but we see no evidence of heterogeneity in Ab staining suggesting that expression is initiated in all hemocytes.

Likewise, for NijA we detect expression in whole larvae preps (circulating hemocytes with SHC contamination) both by RT-PCR as well as using NijA-GFP traps. This expression is reduced in whole larvae preps from clear gut stages with no obvious heterogeneity suggesting this suppression of NijA expression occurs in all hemocytes. Live imaging of the SHCs using the NijA-GFP trap shows that all sessile hemocytes express NijA (Fig. 3K-M).

Our data indicate that all hemocytes express NijA (when SHCs are present) and Ab (when SHCs disperse). NijA is not expressed in a subset of hemocytes that are uniquely targeted to the SHCs, or Ab only in hemocytes that detach from SHCs. Rather all hemocytes have the ability to occupy these niches – if they are available. In response to a reviewer's question on our first submission we alluded to one of the major challenges in the field, the establishment of live imaging systems that would allow live imaging to be performed on SHCs over extended time periods to examine the time course of recruitment of hemocytes into these structures and their dispersal. The SHCs are acutely sensitive to manipulations and changes in environment that make long term, stable imaging of the type required for this experiment very difficult. However, short-term imaging that we performed many years ago (which is buried in supplemental material for Stofanko et al. (2008)) showed hemocytes being recruited into SHCs and detaching into circulation over a period of minutes. While bearing in mind the caveats mentioned above that imaging conditions could be causing hemocytes to detach, we have long thought that while these structures appear static accumulations, they have a high degree of turnover of individual hemocytes from and to circulation.

(4) I may have misread, but the switch on of Abrupt seemed temporal, rather than spatial?
There is temporal regulation of Abrupt in hemocytes with the late third larval ecdysone pulse initiating expression of Abrupt. We do not see any evidence that increased Ab is restricted to subsets of hemocytes as the majority of hemocytes in preparations express Abrupt in clear gut stages.

(5) Are the circulating hemocytes at early stages not adhering because they don't express ninjirin, or for some other reason?
I assume by early stages you mean 1st instar larvae. We have not examined Ninjurin expression in hemocytes at 1st instar stages – partly due to the small number of hemocytes at such stages and low yields for transcriptome or even IF analysis. I suspect Ninjurins are expressed but that a combination of factors could either prevent detection of SHCs (if they occur) or their formation at these stages.

A simple explanation is that as the number of hemocytes contributed to 1st instar larvae from the embryo is low, with hemocyte number considerably fewer than that present at 2nd and 3rd instar stages, SHCs may be difficult to detect simply due to the low numbers of hemocytes that would be present to accumulate in these regions.

However, the fact that we can observe posterior accumulations in 1st instar larvae, but not segmentally repeated SHCs, suggest that we would be able to detect them if present. The more likely explanation for the difference between 1st and later 2nd and 3rd instars is that

© 2024. Published by The Company of Biologists under the terms of the Creative Commons Attribution License (https://creativecommons.org/licenses/by/4.0/).

there are differences in Ninjurin expression between these stages - not in hemocytes but potentially in non-hemocyte tissue underlying the SHCs. In 3rd instar larvae the SHCs occur over non-hemocyte regions that express Ninjurins, and we speculate hemocytes are targeted to these regions by homotypic interaction between Ninjurin on hemocytes and Ninjurins in target tissues. As such, the absence of SHCs in 1st instar larvae could be explained by control of Ninjurin expression in tissue underlying the SHC- absent in 1st instar larvae but appearing in 2nd and 3rd instar. Even if Ninjurins were expressed in hemocytes at all stages, SHCs would therefore be absent in 1st instar larvae.

It is equally feasibly that Ninjurins are one element of a multi-component mechanism that targets hemocytes to SHCs with Ninjurins collaborating with other cell adhesion molecules to stabilize binding. This point is touched on in the response to Reviewer 2: comment 2 below. In this case, while loss of Ninjurins can destabilise binding, the simple presence of Ninjurins alone might be insufficient to cause recruitment at these earlier stages.

(6) *As I finished reading the Results section of this MS, I was expecting to have to suggest that the authors add some text in the Discussion to offer insights beyond Drosophila, but no need. We are told that ninjirins indeed have several interesting functions in mammalian inflammation that will make these findings in the fly even more significant for others.*
We had been careful to avoid "over-speculation" and limited the discussion to that most directly germane to our fly results. However, the reviewer does accurately point out that are a number of publications detailing roles of human Ninjurin 1 (Ninj1) in inflammation, with reports that it acts as a prognostic marker in cardiovascular disease and that peptides that antagonize the N-terminal cell adhesion function of Ninj1 have beneficial impact on disease. Certainly, Kyu-Won Kim's lab have published a number of papers that are suggestive of roles for Ninj1 in controlling leukocyte trafficking into inflamed tissue and function in inflammatory disease. We had included some discussion of these papers in the original draft (3rd to last paragraph of the discussion) in an attempt to highlight similarities between roles for mammalian Ninj1 in leukocyte endothelial transmigration and Drosophila NijA in attachment of hemocytes to SHCs but perhaps failed to make clear the broader implications of the fly results for investigators in mammalian systems. To remedy this, we have altered the text of the 5th paragraph to highlight roles of Ninj1 in leukocyte extravasation and homing, expanded on known examples of Ninj1 function in mammalian leukocyte targeting (for example roles of Ninj1 in leukocyte homing in rat models of experimental autoimmune encephalomyelitis). We have also highlighted potential use of fly systems as models to identify new regulators of these processes.

(7) *Nice paper.*
Thank you!

Reviewer 2:
Major suggestions

(1) *It has been reported that sessile hemocytes detach when vortexing or brushing the larvae. Similarly, hemocytes are expected to disperse upon systemic infection. It would be interesting to see how the hemocytes overexpressing or knocked-down for Ninj behave in these contexts.*
We agree that this would indeed be interesting to see how changes in the levels of NijA alter to properties of SHCs in response to stimuli. As part of the follow-up work to this manuscript, these are exactly the types of experiment that we plan to pursue. As discussed elsewhere in the response (Reviewer 1:comment 3 above) we speculate that features of Ninjurin A encode instability in the adhesive interactions it mediates. We plan to explore the role of the intrinsically disordered domains (IDRs) that comprise the extracellular domain of Ninjurins (especially obvious for NijA) in facilitating protein clustering and whether the NijA IDR mediates the formation of liquid-liquid phase-separated condensates.

We have not discussed this in this manuscript, but we feel it is likely that such phases enable SHC formation and that interesting properties of phases (where small changes in physicochemical conditions cause large shifts in the propensity to form condensates) simultaneously underly the sensitivity of SHCs to perturbation. The type of experiments the reviewer proposes are exactly the types of experiment that can be performed alongside

© 2024. Published by The Company of Biologists under the terms of the Creative Commons Attribution License (https://creativecommons.org/licenses/by/4.0/).

responses to other stressors to establish whether indeed IDR clustering and phase condensates contribute to Ninjurin function.

In comment 5 below the reviewer also references the excellent recent work by the Shim laboratory examining relocalisation of crystal cells and plasmatocytes from circulation under hypoxic conditions. In this publication, Shin and colleagues speculate this relocalisation is accompanied by phase transitions in crystal cell inclusions that liberate oxygen and facilitate to quote, “internal oxygen homeostasis in a process that is comparable to vertebrate respiration”. While the role of crystal cells in oxygenation is intriguing, this manuscript makes clear that hypoxic conditions demonstrably trigger changes in the adhesive properties of hemocytes. From our perspective, to paraphrase the reviewer, it would be even more interesting to see whether these changes in adhesion are encoded in the properties of the NijA IDR. Specifically, does the NijA IDR undergo changes in phase separation in response to oxygen saturation, pH, bacterially derived products and other stimuli?

(2) *The receptors Eater and Multiplexin have been implicated in the formation of sessile patches (Bretscher et al., 2015, Csordars et al., 2020). It is surprising that these important articles are not mentioned and discussed in the present article. Could Ab be involved in the regulation of Eater? How to reconcile the data from these papers and the present observation implying a role of ninjurin?*

Our feeling is roles for NijA and Eater in SHC formation are not mutually-exclusive but probably complementary which would explain why knockdown of either of these factors independently is able to perturb SHCs. It is a false dichotomy that there is an either-or role that needs to be reconciled.

The functions of Eater are clearly complex, spanning a number of processes and a clear mechanistic understanding of Eater function that encompasses all these roles remains to be established. What is known is that Eater was first identified as a factor that mediates bacterial phagocytosis (Kocks et al., 2005) reflecting protein homologies to the broader family of Scavenger-like receptors. Human orthologues of Eater - Clever-1/Stabilin-1/FEEL-1; SCARF1/SREC1; and PEAR1/MEGF12/JEDI – have demonstrated roles in in endocytic trafficking and clearance of a number of substrates. Precisely how these functions intersect with the functions described in Bretscher et al., 2015, Csordars et al., 2020, and observed for some of the human orthologues in leukocyte trafficking (albeit when expressed on endothelium), is not entirely clear.

Data from Bretscher et al., 2015 and Csordars et al., 2020 showing that Eater and Mp potentially interact and are required to maintain SHCs and for mistargeting of hemocytes are interesting and provocative. However, what is perhaps lacking is demonstration of why normal SHCs arise in the defined regions they occupy. If Mp was the only factor mediating hemocyte recruitment to SHCs via interactions with Eater one would assume that SHCs should be prefigured by restricted expression of Mp in regions that underly the normal SHCs, as we see for NijA. Expression of endogenous Mp in larvae is not reported in these manuscripts, making it unclear whether Mp is in fact expressed just where it needs to be to recruit hemocytes to form SHCs, or whether it has generalised expression in the epidermis and needs additional factors like NijA for example to establish the restricted pattern. Some suggestion that the situation may be more complex is that, although clonal over-expression of Mp in the fat body causes hemocyte mistargeting, interestingly not all Mp over-expressing cells recruit hemocytes (Csordars et al., 2020; Fig. 6B) suggesting that there are additional factors at play which we would suggest are Ninjurins.

Setting aside the caveats listed above, a compromise explanation is that hemocyte recruitment probably depends on a number of complimentary factors that work together to ensure stable recruitment of hemocytes to the SHCs (which could rely on both homotypic and heterotypic interactions), clustering of hemocytes within SHCs (which would rely on homotypic interactions) and control of the timing and location of SHC formation. To reflect this, we have added the following text to the Discussion section (4th paragraph): “*While our manuscript highlights the role of Ninjurin-mediated homotypic interactions in maintaining SHCs it is important to acknowledge that other factors, such as heterotypic interactions*

© 2024. Published by The Company of Biologists under the terms of the Creative Commons Attribution License (https://creativecommons.org/licenses/by/4.0/).

between the transmembrane protein Eater and collagen proteoglycans, have also been proposed to regulate SHCs (Csordás et al., 2020). Knockdown of either NijA or Eater results in loss of larval SHCs suggesting that the final stereotyped pattern of SHCs may arise from an interplay between both heterophilic Eater-mediated matrix interactions, and homophilic NijA-mediated interactions between NijA-expressing hemocytes and spatially restricted non-hemocyte-expressed NijA."

To address the question on regulation: we have no evidence that Ab or NijA regulate the Eater/Mp axis. While analysis of Eater transcript levels indicates reduction in white prepupae (Kocks et al., 2005), which mirrors the effects of Ab on NijA expression, our analysis of Ab ChiP-seq data do not support direct regulation of Eater by Ab. It is, however, possible that some of the other factors we show co-operate with Ab to control NijA, for example Blimp-1, may participate in this repression.

(3) *A study analyzing the role of Ninjurin A (Broderick et al., 2012) is not mentioned in apoptosis. It should be discussed.*
Broderick et al 2012 is consistent with recent findings on human Ninj1 clearly demarcating the role of Ninjurins in facilitating pyroptosis. As the recent manuscripts clearly define function of Ninjurins in pyroptosis while Broderick and co-workers simply reported non-apoptotic cell death functions, we had prioritized discussion of the more recent publications on human Ninjurins that reveal mechanism. However, as results of Broderick et al 2012 can now be interpreted in light of the recent mammalian Ninj1 papers as reflecting pyroptosis, we have included a citation to this manuscript in the discussion text where the pyroptotic function of Ninjurins is described. Specifically, we have added the following text "*Finally, while our report has focused on the cell adhesion functions of Ninjurins, it is also important to consider that recent data implicates human Ninj1 in pyroptosis, agreeing with previous observations in Drosophila showing NijA is required for non-apoptotic cell death (Broderick et al., 2012).*"

(4) *The NijA null mutant should be used to address the role of NijA in hemocyte sessility*
Our data suggests that NijA is targeted to sessile compartments through homophilic interactions between NijA expressed on hemocytes and NijA expressed in non-hemocytic tissues that associate with the SHCs. The advantage of using GAL4-targeted inducible RNAi to test function, as we done here, is that we have been able to specifically ablate NijA expression in hemocytes to show that hemocyte-expressed NijA is required for SHC formation. While we do not have an equivalent GAL4 driver that expresses solely in the associated non-hemocyte tissue to perform the reciprocal experiment (knock out NijA in non-hemocyte tissue and show loss of SHCs) use of GAL4 inducible RNA does allow us to specifically remove NijA in hemocytes while leaving other tissues unaffected. In contrast, use of NijA null mutants would affect expression in both tissues making it difficult to determine whether loss of SHCs is due to effects on NijA expression in hemocytes, in non-hemocyte tissue associated with the SHC, in both tissues or potentially through indirect effects of loss of NijA in a 3rd tissue that are transmitted to hemocytes.

Minor suggestions

(5) *The recent study showing the migration of hemocytes, notably crystal cells, to sessile patches upon H2O2 should be mentioned (Shin et al., Nature 2024). Thus, sessile patches could have a role in hemocyte/hemolymph oxygenation.*
Thank you for highlighting the very interesting manuscript of Shin and colleagues that was published while our manuscript was under review. This is an extremely interesting manuscript and the response to low oxygen tension is certainly illuminating. As described in the response to Reviewer 2: point 1 above this may have impact on controlling the adhesive properties of Ninjurins. We have included this citation in the discussion where we describe a model for controlling Ninjurin function in response to stimuli. Specifically: we have added the following text to the Discussion: "*We speculate that the regulation of Ninjurin expression and activity allows a wide range of stimuli to be integrated, enabling the release of hemocytes in response to a variety of signals. These include developmental signals like ecdysone as we have shown here (Fig. 7D-E), as well as immune challenges (Fig. 7F) and potentially even chemical signals such as oxygen tension (Shin et al., 2024).*"

© 2024. Published by The Company of Biologists under the terms of the Creative Commons Attribution License (https://creativecommons.org/licenses/by/4.0/).

(6) *Top of page 5: what are da neurons?*
Dendritic arborization da neurons are segmentally repeated PNS sensory neurons that occupy stereotyped positions under the larval cuticle, and which also exhibit characteristic differences (depending on position) in the extent of arborization of their dendritic tress, from class I having simple arbor and class IV complex arbors. Abrupt was identified in genetic screens for switching arborization states of these neurons. DOI:10.1146/annurev.neuro.29.051605.112907 provides a good general review of these neurons.

(7) *Bottom of page 7 'determining that each compartment' Explicit what are those compartments.*
We have changed this text to read "*determining that the largest posterior SHC*"

(8) *Page 13: Stofanko is not the only ref revealing that hemocytes leave sessile patches upon challenge.*
Indeed. The context of this citation is building on our previous speculation "We had previously speculated that dorsal SHCs provide a reservoir where hemocytes are held latent in the absence of immune challenge …. (Stofanko et al., 2008)". Zettervall and colleagues (2004) showed loss of sessile compartments following wasp infestation with the authors speculating that "It is possible that these sessile hemocytes act as a source of lamellocyte precursors". One could argue that this is subtly different in meaning but we have added a citation to this reference, which is already cited elsewhere in the manuscript, at this location. Text has been modified to read: "*It has previously been speculated that dorsal SHCs provide a reservoir where hemocytes are held latent in the absence of immune challenge but from which they can be deployed into circulation to deal with challenge (Stofanko et al., 2008; Zettervall et al., 2004).*"

(9) *Page 15: Add Stephenson et al., 2022 to indicate that hemocyte are critical during metamorphosis*
Lanot and colleagues (2001) is supportive of roles of hemocytes in pupal tissue remodeling and has the added benefit of being a transformative publication in the field, both characterising sessile hemocyte compartments (they termed them sessile islets) and showing hemocytes involved in "*phagocytosis of histolysing larval tissue*" at pupal stages. However, I have added the more recent suggested reference and modified the text in the discussion to read accordingly "*However, larval hemocyte populations undergo similar developmental remodelling with larval hemocytes contributing vital functions to immunity and tissue remodeling during pupal stages (Lanot et al., 2001; Stephenson et al., 2022).*"

(10) *There is no method section describing the statistics used in this study. It is unclear how the t-test was applied on different categories (number of patches) at the same time. A different test might be more suited. There is no statistical analysis on Fig2H. The Y axis legend is missing in Fig2M.*
We have revisited the statistical tests used in the study and where appropriate re-analysed data using either One-way ANOVA with Tukey's post-hoc test to perform pairwise comparisons (Fig. 2H, 2H, 6C) or Kruskal-Wallis test with Dunn's post-hoc test to perform pairwise comparisons for SHC ("number of patches") data comparisons (Fig. 2M, 2N, 4G, 4H, 4Q and 6E). Descriptions of tests and values returned have been added to the respective figure legends. We have also added a section to the materials and methods that details these procedures - "*Scoring of dorsal sessile compartments and statistical analysis*". This section incorporates the following text describing SHC statistical tests: specifically, "*A Kruskal-Wallis test, a non-parametric alternative to one-way ANOVA, was conducted to determine statistically significant differences in SHC numbers across genotypes and stages (wandering versus clear gut) for each experiment. Dunn's post hoc tests were conducted to test pairwise comparisons and identify specific groups that differ significantly*".

(11) *Survival to bacterial infection can vary drastically across genetic backgrounds. Therefore, it is necessary to also test the UAS line crossed to + in the infection experiments.*
This was performed alongside the GAL4 driver cross with w[1118]. However, we observed no statistically significant differences between GAL4 x w[1118] and UAS x w[1118] crosses in survival and just presented the GAL4 x w[1118] survival curve for simplicity (felt the

© 2024. Published by The Company of Biologists under the terms of the Creative Commons Attribution License (https://creativecommons.org/licenses/by/4.0/).

figures were already quite busy). To address this, we have added the UAS-NijA x w[1118] curves to a new Fig. 7A-C.

(12)It is stated that gem overexpression has no impact on hemocyte homing but Figure 6E shows a significant difference, could you explain this discrepancy?
Yes, this statement is incorrect. Gem overexpression does have an effect although its effect at wandering stages is milder than that observed with Ab or Blimp-1. We have clarified this is the relevant results section, specifically changing text to read "*Reciprocally, over-expression of these TFs (except for ewg that could not be assayed) disrupted dorsal SHCs with strongest effects observed with Blimp-1 (Fig. 6E, compare Cont with OE panels). Significantly though, over-expression phenotypes were milder than Ab over-expression phenotypes suggesting that Ab plays the dominant role in triggering hemocyte dispersal.*"

(13)Purified LPS does not activate the Drosophila immune response, therefore it is misleading to discuss together response to LPS and bacterial infection (see Leulier et al., 2003, Kaneko et al., 2004). This section might be rephrased to avoid confusion.
Apologies, it is of course true that LPS does not activate *Drosophila* immune signaling cascades and transcriptional responses. There is however data from Kyu-Won Kim's laboratory indicating that LPS can bind to Ninj1. They were able to identify the region that binds LPS (aa 81-100) which corresponds to the first transmembrane helix of Ninj1. This region is conserved with Ninjurin A suggesting that NijA might also share the ability to bind LPS. To resolve the confusion, we have rephrased this part of the discussion. Specifically altering the section detailing microarray analysis and NijA to remove reference to LPS: "*However, previous publications show that NijA expression and activity can be influenced by a number of additional stimuli. NijA transcription is responsive to immune triggers (Boutros et al., 2002; Buchon et al., 2009), while immune challenge can activate Mmp1 (Johansson et al., 2005) which, in turn, can cleave and regulate Ninjurins (Jeon et al., 2020; Zhang et al., 2006), allowing hemocyte adhesion to be disrupted. In mammalian systems, LPS can bind human Ninj1 (Shin et al., 2016) and has the potential to disrupt ectodomain function and homophilic cell adhesion.*" Hopefully this addresses any unintended confusion regarding LPS and immune signaling in flies.

<u>Second decision letter</u>

MS ID#: DEVELOP/2024/202977

MS TITLE: Abrupt-mediated control of Ninjurins regulates Drosophila sessile hemocyte compartments

AUTHORS: So Yeon Kwon, Kimberley Chan, Martin Stofanko, Ka Hei Chan, and Paul Badenhorst

I am happy to tell you that your manuscript has been accepted for publication in Development, pending our standard publication integrity checks.

© 2024. Published by The Company of Biologists under the terms of the Creative Commons Attribution License (https://creativecommons.org/licenses/by/4.0/).
